# Supplementary material for: Structural insights into the interplay between microtubule polymerases, γ-tubulin complexes and their receptors
Source: Nat Commun. 2025 Jan 5;16:402. doi: 10.1038/s41467-024-55778-7 (PMC11701102; doi:10.1038/s41467-024-55778-7)
Supplement: Supplementary file 1 — Supplementary Information [file 41467_2024_55778_MOESM1_ESM.pdf]

Supplementary Information for

Structural insights into the interplay between microtubule polymerases, γ-tubulin complexes and their receptors

Anjun Zheng[1,*], Bram J.A. Vermeulen[1,*], Martin Würtz[1,2,*], Annett Neuner[1], Nicole Lübbehusen[1], Matthias P. Mayer[1], Elmar Schiebel[1,#] and Stefan Pfeffer[1,#]

[1] Zentrum für Molekulare Biologie der Universität Heidelberg (ZMBH), Germany

[2]European Molecular Biology Laboratory (EMBL), Heidelberg Meyerhofstraße 1, 69117 Heidelberg, Germany

* These authors contributed equally

[#] All correspondence should be addressed to Stefan Pfeffer (s.pfeffer@zmbh.uni-heidelberg.de) or Elmar Schiebel (e.schiebel@zmbh.uni-heidelberg.de)

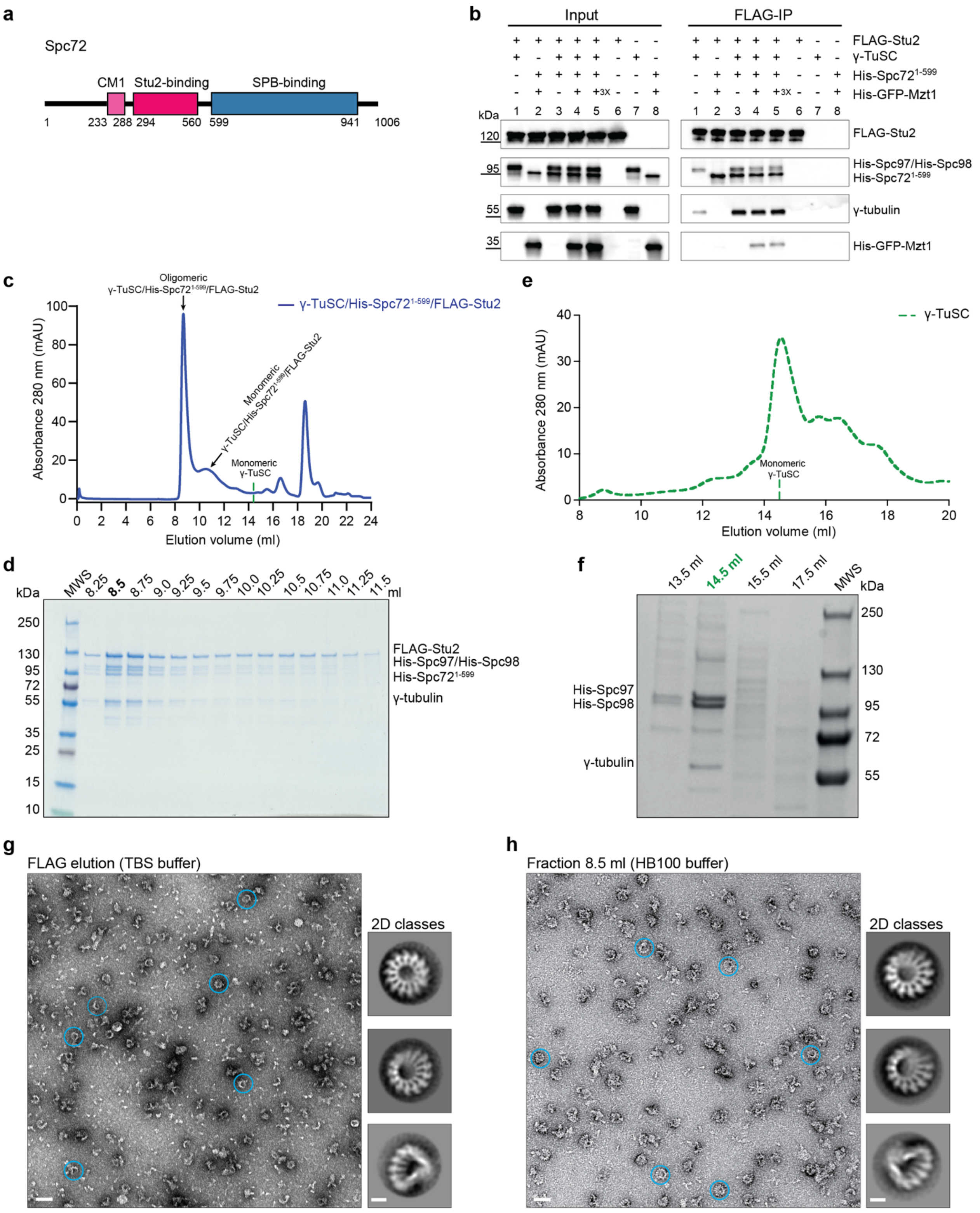
a
Spc72
CM1
Stu2-binding
SPB-binding
1 233 288 294 560 599 941 1006
b
Input
FLAG-IP
FLAG-Stu2
γ-TuSC
His-Spc72^1-599
His-GFP-Mzt1
kDa
120
95
55
35
His-Spc97/His-Spc98
His-Spc72^1-599
γ-tubulin
His-GFP-Mzt1
c
Oligomeric γ-TuSC/His-Spc72^1-599/FLAG-Stu2
Monomeric γ-TuSC/His-Spc72^1-599/FLAG-Stu2
Monomeric γ-TuSC
Absorbance 280 nm (mAU)
Elution volume (ml)
e
γ-TuSC
Monomeric γ-TuSC
Absorbance 280 nm (mAU)
Elution volume (ml)
d
kDa
MWS
ml
FLAG-Stu2
His-Spc97/His-Spc98
His-Spc72^1-599
γ-tubulin
f
13.5 ml
14.5 ml
15.5 ml
17.5 ml
MWS
kDa
His-Spc97
His-Spc98
γ-tubulin
g
FLAG elution (TBS buffer)
2D classes
h
Fraction 8.5 ml (HB100 buffer)
2D classes

Supplementary Figure 1. *Candida albicans* Stu2, Spc72$^{1\text{-}599}$ and γ-TuSC co-purify and form ring-like oligomers.

(a) Scheme of Spc72 with different functional domains as identified in *S. cerevisiae* [1] is depicted in different colours; approximate domain boundaries are indicated. **(b)** FLAG-Stu2 immunoprecipitations show that Stu2 interacts with the γ-TuSC (lane 1) and His-Spc72$^{1\text{-}599}$ (lane 2) individually and together (lane 3). Moreover, γ-TuSC, His-Spc72$^{1\text{-}599}$, and His-GFP-Mzt1 can all exist in a complex with Stu2 (lanes 4 and 5). Anti-FLAG antibody against Stu2, anti-His antibody against His-Spc97/His-Spc98/His-Spc72$^{1\text{-}599}$/His-GFP-Mzt1, and anti-γ-tubulin antibodies were used to visualise the proteins. $N = 2$ biologically independent experiments. Corresponding input and FLAG IP lanes were labelled with the same number to aid comparison. **(c)** Size-exclusion chromatography (SEC, Superose 6 increase 10/300GL) was used to analyse the oligomeric state of complexes in the γ-TuSC/Spc72$^{1\text{-}599}$/FLAG-Stu2 sample. Elution volume of free monomeric γ-TuSC is indicated at 14.5 ml (green line, see panels (e), (f)). $N = 3$ biologically independent experiments. **(d)** The protein content of different fractions (8.25-11.5 ml) after SEC of the γ-TuSC/His-Spc72$^{1\text{-}599}$/FLAG-Stu2 sample (shown in (c)) was visualised using Coomassie-stained SDS-PAGE. $N = 3$ biologically independent experiments. **(e)** SEC chromatogram of free monomeric γ-TuSC (see methods), with its peak location marked as a green dashed line. $N = 3$ biologically independent experiments. **(f)** The protein content of different fractions after SEC of free monomeric γ-TuSC (shown in (e)) was visualised using Coomassie-stained SDS-PAGE. $N = 3$ biologically independent experiments. (**g, h**) Negative stain EM micrographs and 2D classes of the γ-TuSC/His-Spc72$^{1\text{-}599}$/FLAG-Stu2 complex as obtained through FLAG-Stu2 purification (g) or SEC (h; 8.5 ml fraction from (c)). Scale bars represent 50 nm for the micrographs and 10 nm for the 2D class averages. $N = 3$ biologically independent experiments. Source data are provided in the Source Data file.

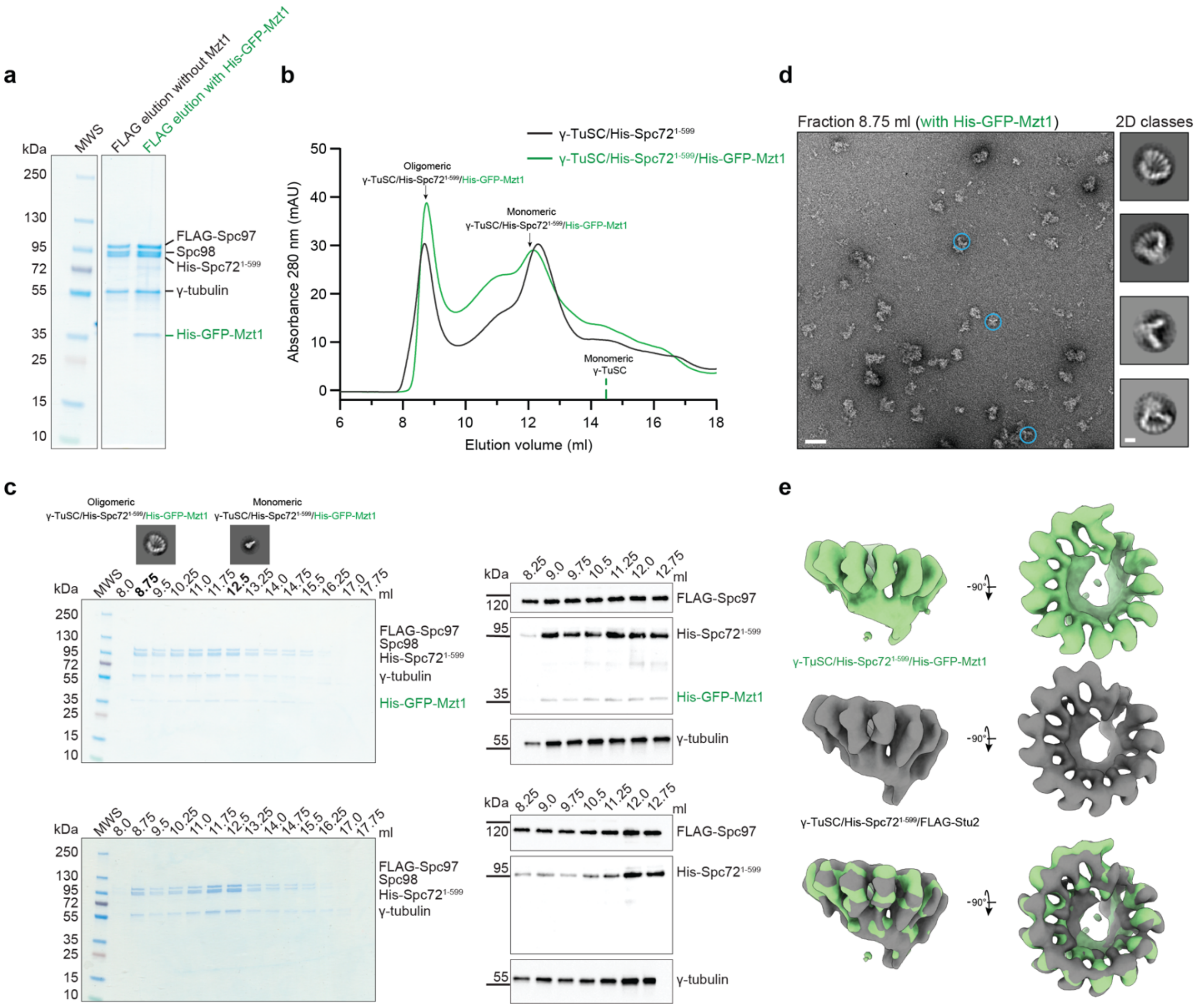

Supplementary Figure 2. Co-expression of Mzt1 has no impact on oligomer formation and the overall structure of γ-TuSC oligomers.

(a) Coomassie-stained SDS-PAGE gel of FLAG-purified γ-TuSC/His-Spc72$^{1\text{-}599}$ complexes with and without His-GFP-Mzt1. **(b)** SEC chromatograms of γ-TuSC/His-Spc72$^{1\text{-}599}$ complexes with (black) and without (green) co-expression of His-GFP-Mzt1. Fractions corresponding to monomeric and oligomeric γ-TuSC/His-Spc72$^{1\text{-}599}$ with or without His-GFP-Mzt1 are indicated. $N = 2$ biologically independent experiments. **(c)** The protein content of fractions from SEC profiles shown in panel (b) was visualised by Coomassie-staining and Western blotting. Representative negative stain 2D classes for monomeric and oligomeric γ-TuSC are shown. Western blotting was performed with antibodies against FLAG-Spc97, His-Spc72$^{1\text{-}599}$/His-GFP-Mzt1 and γ-tubulin. $N = 2$ biologically independent experiments. **(d)** Negative stain EM micrograph and 2D classes of the γ-TuSC/His-Spc72$^{1\text{-}599}$ complex co-expressed with His-GFP-Mzt1 from the SEC fraction at 8.75 ml (see panels b and c). Scale bars represent 50 nm for the micrograph and 10 nm for the 2D class averages. **(e)** Negative stain 3D class averages of γ-TuSC rings formed after co-expression with His-Spc72$^{1\text{-}599}$/His-GFP-Mzt1 (green) or His-Spc72$^{1\text{-}599}$/FLAG-Stu2 (grey), showing the overall geometry of the ring is unaffected by the co-expression of His-GFP-Mzt1. His-GFP-Mzt1 dissociation during EM grid

preparation cannot be entirely excluded, because no additional density attributable to Mzt1 can be observed. $N = 1$ biologically independent experiment. Source data are provided in the Source Data file.

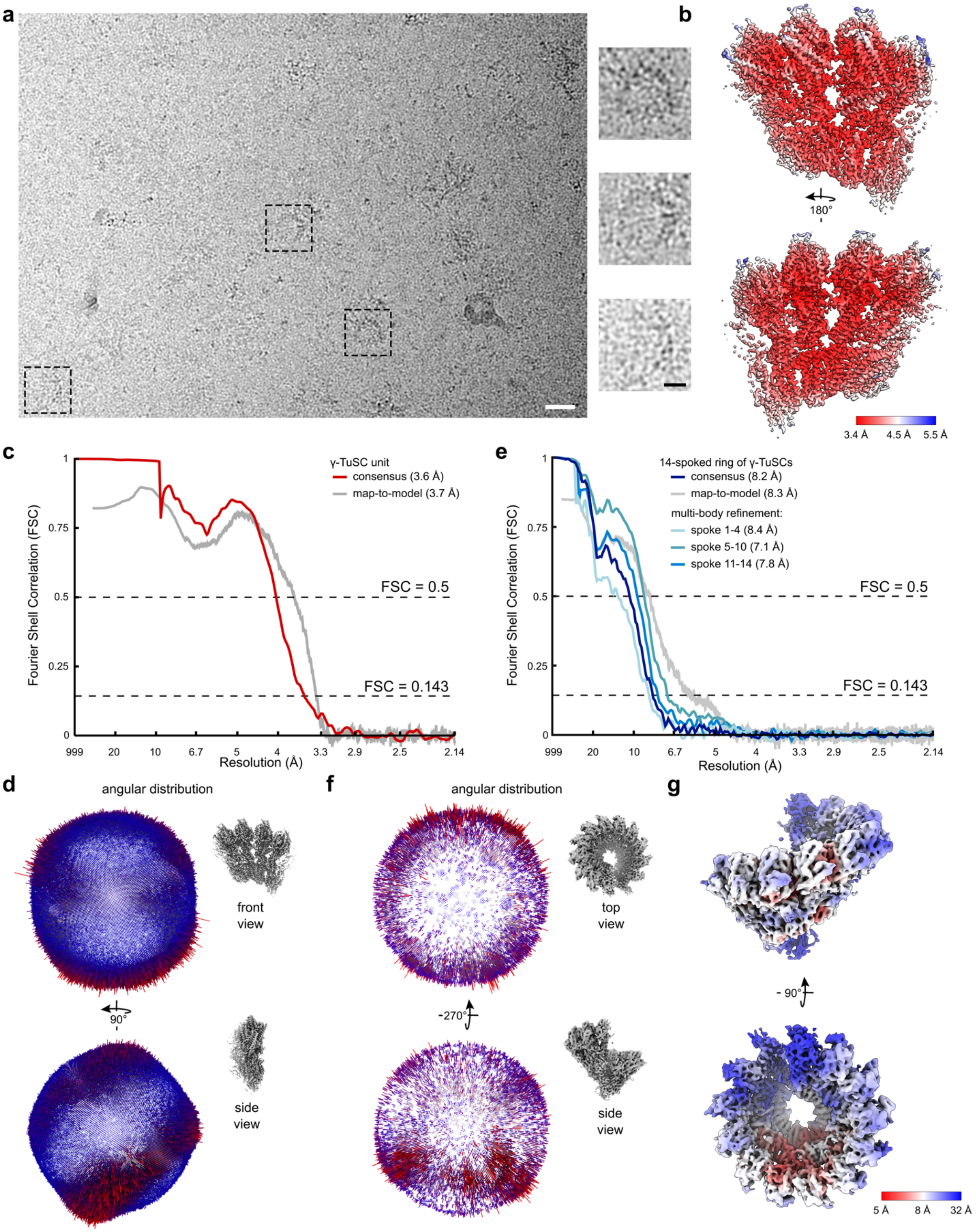
a
b
180°
3.4 Å
4.5 Å
5.5 Å
c
γ-TuSC unit
consensus (3.6 Å)
map-to-model (3.7 Å)
FSC = 0.5
FSC = 0.143
Fourier Shell Correlation (FSC)
Resolution (Å)
e
14-spoked ring of γ-TuSCs
consensus (8.2 Å)
map-to-model (8.3 Å)
multi-body refinement:
spoke 1-4 (8.4 Å)
spoke 5-10 (7.1 Å)
spoke 11-14 (7.8 Å)
FSC = 0.5
FSC = 0.143
Fourier Shell Correlation (FSC)
Resolution (Å)
d
angular distribution
front view
90°
side view
f
angular distribution
top view
-270°
side view
g
- 90°
5 Å
8 Å
32 Å

Supplementary Figure 3. Example micrograph and resolution estimates of cryo-EM reconstructions.

(a) Cut-out from an example cryo-EM micrograph of ring-like γ-TuSC/His-Spc72$^{1\text{-}599}$ complexes purified via FLAG-Stu2. Exemplary particles are indicated and shown enlarged to the right. Micrograph was low-pass filtered to 20 Å. Scale bars represent 25 nm (micrograph) and 10 nm (particles). **(b)** Reconstruction of a γ-TuSC unit within a higher oligomer coloured according to the local resolution estimate. **(c)** Fourier shell correlation (FSC) between two independently refined half-set reconstructions of the γ-TuSC unit within a higher oligomer. Map-to-model FSC curve is overlaid. The resolution value at which the FSC crosses FSC=0.143 (independently refined half-set reconstruction) or FSC=0.5 (map-to-model correlation) is indicated. Colouring as indicated. **(d)** Angular distribution of particle views of the γ-TuSC unit within a higher oligomer. Orientations as indicated. **(e)** Fourier shell correlation (FSC) between two independently refined half-set reconstructions of the 14-spoked ring of γ-TuSC units (d), after consensus and multi-body refinements. Map-to-model FSC curve is overlaid. The resolution value at which the FSC crosses FSC=0.143 (independently refined half-set reconstructions) or FSC=0.5 (map-to-model correlation) is indicated. Colouring as indicated. **(f)** Angular distribution of particle views of the 14-spoked ring of γ-TuSC units. Orientations as indicated. **(g)** Reconstruction after multi-body refinement of the 14-spoked ring of γ-TuSC units coloured according to the local resolution estimate. In panel (f) and (g), dust is hidden.

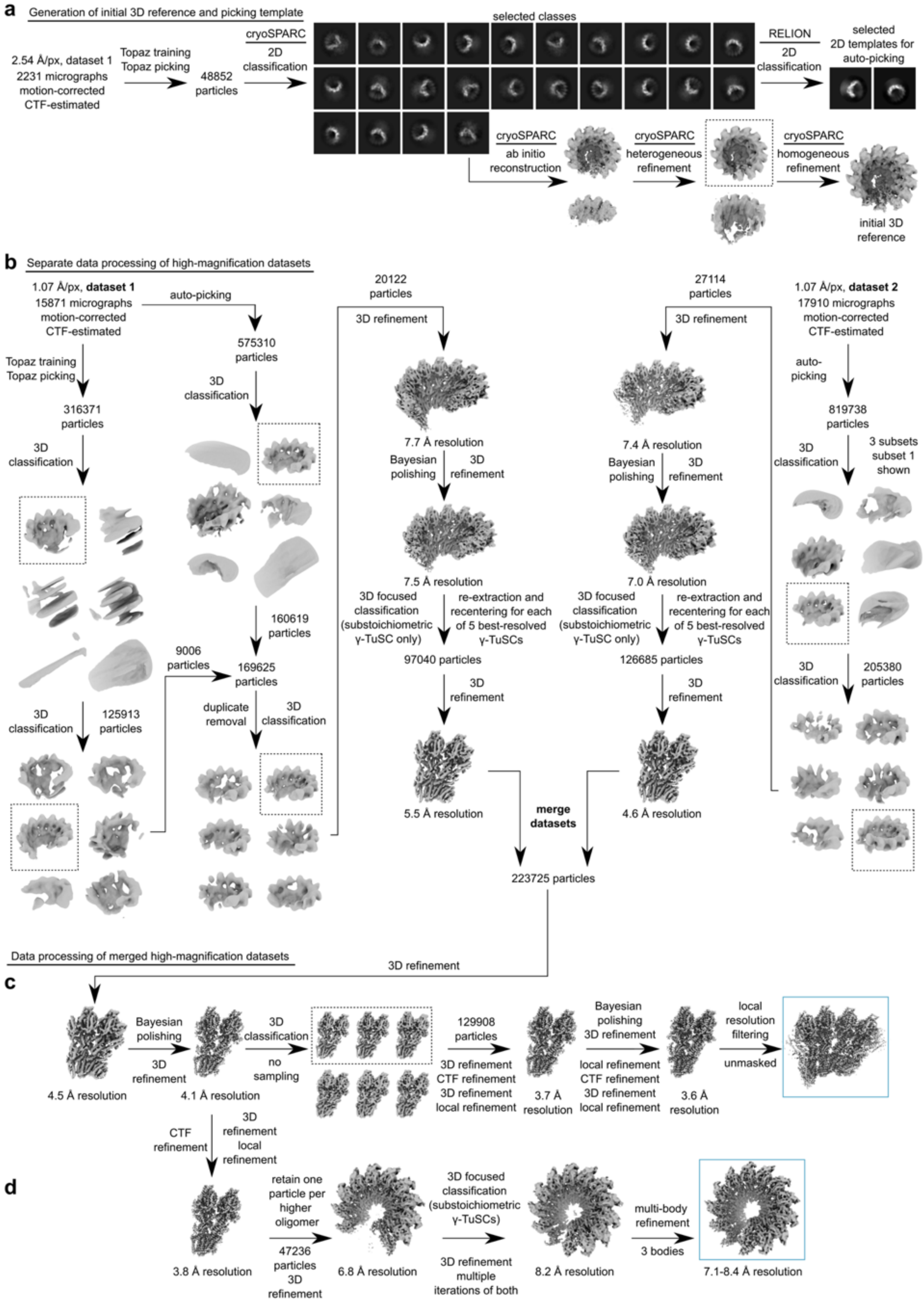

a
Generation of initial 3D reference and picking template
2.54 Å/px, dataset 1
2231 micrographs
motion-corrected
CTF-estimated
Topaz training
Topaz picking
48852 particles
cryoSPARC
2D classification
selected classes
RELION
2D classification
selected 2D templates for auto-picking
cryoSPARC
ab initio reconstruction
cryoSPARC
heterogeneous refinement
cryoSPARC
homogeneous refinement
initial 3D reference
b
Separate data processing of high-magnification datasets
1.07 Å/px, dataset 1
15871 micrographs
motion-corrected
CTF-estimated
auto-picking
575310 particles
Topaz training
Topaz picking
316371 particles
3D classification
3D classification
160619 particles
9006 particles
169625 particles
duplicate removal
3D classification
125913 particles
20122 particles
3D refinement
7.7 Å resolution
Bayesian polishing
3D refinement
7.5 Å resolution
3D focused classification (substoichiometric γ-TuSC only)
re-extraction and recentering for each of 5 best-resolved γ-TuSCs
97040 particles
3D refinement
5.5 Å resolution
merge datasets
27114 particles
3D refinement
7.4 Å resolution
Bayesian polishing
3D refinement
7.0 Å resolution
3D focused classification (substoichiometric γ-TuSC only)
re-extraction and recentering for each of 5 best-resolved γ-TuSCs
126685 particles
3D refinement
4.6 Å resolution
1.07 Å/px, dataset 2
17910 micrographs
motion-corrected
CTF-estimated
auto-picking
819738 particles
3D classification
3 subsets subset 1 shown
3D classification
205380 particles
223725 particles
Data processing of merged high-magnification datasets
3D refinement
c
4.5 Å resolution
Bayesian polishing
3D refinement
4.1 Å resolution
3D classification
no sampling
129908 particles
3D refinement
CTF refinement
3D refinement
local refinement
3.7 Å resolution
Bayesian polishing
3D refinement
local refinement
CTF refinement
3D refinement
local refinement
3.6 Å resolution
local resolution filtering
unmasked
CTF refinement
3D refinement
local refinement
d
3.8 Å resolution
retain one particle per higher oligomer
47236 particles
3D refinement
6.8 Å resolution
3D focused classification (substoichiometric γ-TuSCs)
3D refinement
multiple iterations of both
8.2 Å resolution
multi-body refinement
3 bodies
7.1-8.4 Å resolution

Supplementary Figure 4. Cryo-EM data processing scheme of ring-like γ-TuSC/Spc72$^{1-599}$ complexes purified via FLAG-Stu2.

(a) An initial dataset at intermediate magnification was used to generate a 2D template for particle picking and an initial 3D reference density for 3D classification. **(b-d)** Two higher magnification datasets were first processed independently and merged to resolve the structure of the γ-TuSC unit within higher oligomers to 3.6 Å (c), as well as the full ring of seven γ-TuSC units to 8.2 Å (d). Unless otherwise indicated, data processing was performed in RELION.

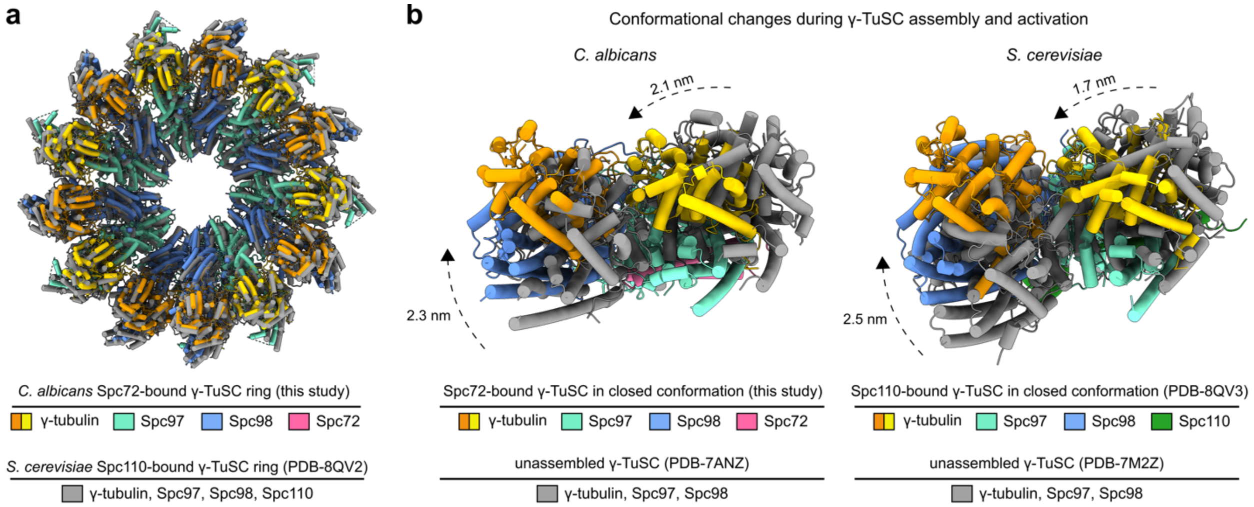

Supplementary Figure 5. Comparison of conformational changes during *C. albicans* Spc72$^{1-599}$- and *S. cerevisiae* Spc110-induced γ-TuSC oligomerisation and activation.

(a) Comparison of the Spc72$^{1-599}$-bound *C. albicans* γ-TuSC ring with the MT-capping Spc110-bound *S. cerevisiae* γ-TuSC ring (PDB-8QV2)[2]. Models were superposed on all γ-tubulin molecules. Colouring scheme as indicated. **(b)** Comparison of isolated γ-TuSCs (grey models) versus assembled and closed γ-TuSCs (coloured models) in complex with *C. albicans* Spc72$^{1-599}$ (left, this study and PDB-7ANZ[3]) or *S. cerevisiae* Spc110 (right, PDB-8QV3[2] and PDB-7M2Z[4]). Colouring scheme as indicated. Models were superposed on N-terminal part of GRIP1 domains for both spokes. Indicated distances are calculated using Gln12 of the γ-tubulin molecule at the respective spoke.

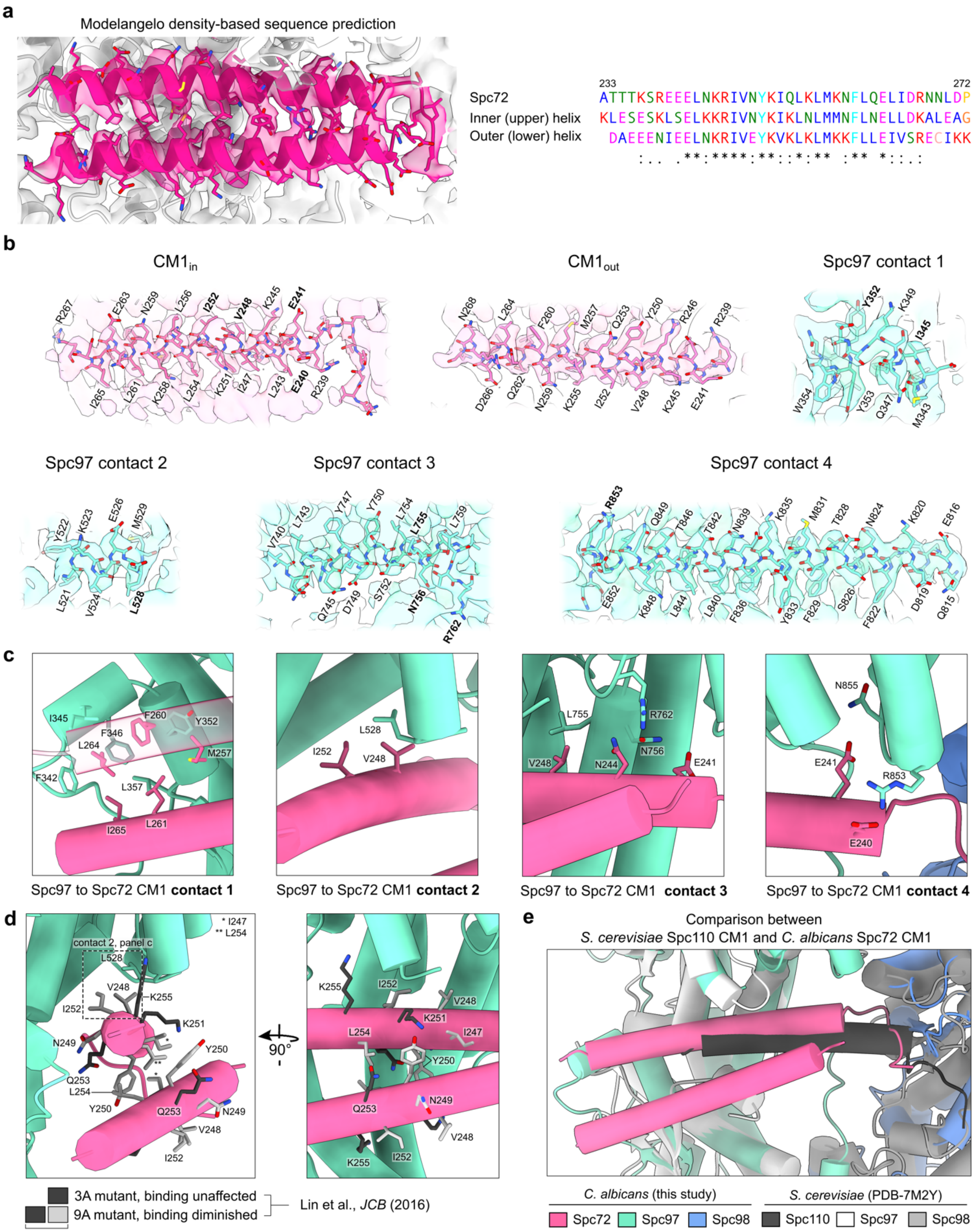

a
Modelangelo density-based sequence prediction
233
272
Spc72
ATTTKSREEELNKRIVNYKIQLKLMKNFLQELIDRNNLDP
Inner (upper) helix
KLESESKLSELKKRIVNYKIKLNLMMNFLNELLDKALEAG
Outer (lower) helix
DAEEENIEELNKRIVEYKVKLKLMKKFLLEIVSRECIKK
b
CM1in
CM1out
Spc97 contact 1
Spc97 contact 2
Spc97 contact 3
Spc97 contact 4
c
Spc97 to Spc72 CM1 contact 1
Spc97 to Spc72 CM1 contact 2
Spc97 to Spc72 CM1 contact 3
Spc97 to Spc72 CM1 contact 4
d
* I247
** L254
contact 2, panel c
90°
3A mutant, binding unaffected
9A mutant, binding diminished
Lin et al., JCB (2016)
e
Comparison between
S. cerevisiae Spc110 CM1 and C. albicans Spc72 CM1
C. albicans (this study)
S. cerevisiae (PDB-7M2Y)
Spc72
Spc97
Spc98
Spc110
Spc97
Spc98

Supplementary Figure 6. Detailed structural analysis of dimeric CM1 motif binding in *Candida albicans*.

(a) Top: zoom into the coiled-coil density on the back of Spc97 with the atomic model built by sequence-free mode of ModelAngelo superposed. Bottom: sequence alignment for the Spc72 CM1 motif and the coiled coil helices as predicted by ModelAngelo. **(b)** Fit of atomic model to the cryo-EM reconstruction for secondary structure elements involved in the interactions shown in panel (c). Residues shown in panel (c) are highlighted in bold. Unmasked and unsegmented reconstructions are shown. Plane clipping in UCSF ChimeraX[5] was used to allow for an unobstructed view on the fitted atomic model. Colouring as in panel (c). **(c)** The CM1 motif of Spc72 (pink) contacts Spc97 (light blue) through four interaction elements. Residues involved are labeled. **(d)** Visualisation of Spc72 CM1 motif residues that were mutated by Lin et al. [6]. Consistent with their affinity measurements, some of the residues modified in the γ-TuSC binding-deficient 9A mutant (coloured in light and dark grey) contact the γ-TuSC and are involved in the dimerisation interface of the coiled coil. Conversely, none of the residues in the γ-TuSC binding-competent 3A mutant (dark grey) of Spc72 are in direct contact with the γ-TuSC or involved in the coiled-coil dimerisation interface. Colouring as in panel (c) unless otherwise mentioned. **(e)** Comparison of the binding mode of the dimeric *C. albicans* Spc72 CM1 motif and the monomeric *S. cerevisiae* Spc110 CM1 motif[4] (PDB-7M2Y, EMD-23637) at the inter-γ-TuSC interface. Models were superimposed on Spc97 and Spc98. Colouring as indicated.

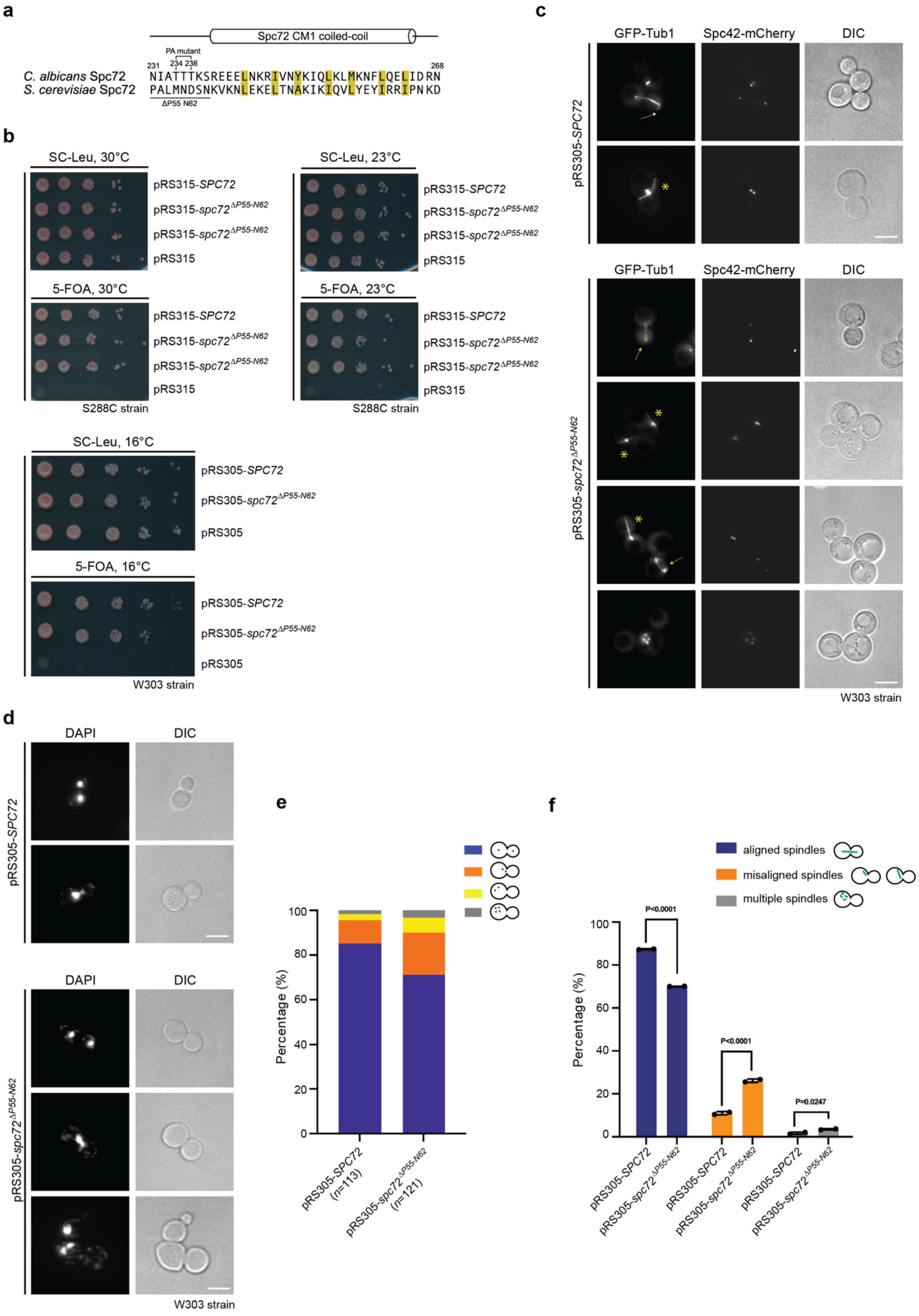

a
Spc72 CM1 coiled-coil
PA mutant
231 234 236 268
C. albicans Spc72 NIATTTKSREEELNKRIVNYKIQLKLMKNFLQELIDRN
S. cerevisiae Spc72 PALMNDSNKVKNLEKELTNAKIKIQVLYEYIRRIPNKD
ΔP55 N62
b
SC-Leu, 30°C
5-FOA, 30°C
SC-Leu, 23°C
5-FOA, 23°C
pRS315-SPC72
pRS315-spc72ΔP55-N62
pRS315
S288C strain
SC-Leu, 16°C
5-FOA, 16°C
pRS305-SPC72
pRS305-spc72ΔP55-N62
pRS305
W303 strain
c
GFP-Tub1
Spc42-mCherry
DIC
pRS305-SPC72
pRS305-spc72ΔP55-N62
W303 strain
d
DAPI
DIC
pRS305-SPC72
pRS305-spc72ΔP55-N62
W303 strain
e
Percentage (%)
pRS305-SPC72 (n=113)
pRS305-spc72ΔP55-N62 (n=121)
f
aligned spindles
misaligned spindles
multiple spindles
Percentage (%)
P<0.0001
P<0.0001
P=0.0247
pRS305-SPC72
pRS305-spc72ΔP55-N62

Supplementary Figure 7. *In vivo* analysis of *spc72*$^{\Delta P55\text{-}N62}$ function in budding yeast.

(a) Sequence alignment of the Spc72 CM1 motifs in *C. albicans* and *S. cerevisiae* with hydrophobic residues at the dimerisation interface highlighted in yellow. *C. albicans* Spc72$^{1\text{-}599,PA}$ mutant residues (T234 and T236, respectively) as well as residues (P55-N62) deleted to abolish Spc72-Spc98 interaction in *S. cerevisiae* are indicated. **(b)** Cell viability test of the *spc72*$^{\Delta P55\text{-}N62}$ mutant in *S. cerevisiae*. Cells were serially diluted and grown at the indicated temperatures. $N = 3$ biologically independent experiments. **(c)** *SPC72* and *spc72*$^{\Delta P55\text{-}N62}$ cells (W303 strain background) were cultured on 5-FOA plates at 16°C and observed by microscopy, using GFP-Tub1 (marking MTs) and Spc42-mCherry (marking the SPB) for visualisation. cMTs are indicated by white arrows, defective cMTs by yellow arrows, and elongated cMTs are by yellow asterisks. Scale bar: 5 µm. DIC: differential interference contrast. $N = 2$ biologically independent experiments. **(d)** DAPI staining analysis of *SPC72* and *spc72*$^{\Delta P55\text{-}N62}$ cells (W303 strain background) cultured at 16°C. Scale bar: 5 µm. $N = 2$ biologically independent experiments. **(e)** Quantification of nuclear phenotypes for *SPC72* and *spc72*$^{\Delta P55\text{-}N62}$ cells from (d) shown as bar plot (n=113 cells for *SPC72* and n=121 for *spc72*$^{\Delta P55\text{-}N62}$ cells). In *SPC72* cells or *spc72*$^{\Delta P55\text{-}N62}$, 85.1% or 71.2% of cells display normal separation of nuclei (dark blue), 10.5% or 18.8% of cells have their nuclei near the bud neck but still localised at the mother side (orange), 2.7% or 6.7% of cells have the nuclei in the mother cell body (yellow) and 1.7% or 3.3% of cells have multiple nuclei (grey), respectively. $N = 2$ biologically independent experiments quantified together. **(f)** Quantification of MT phenotypes of *SPC72* and *spc72*$^{\Delta P55\text{-}N62}$ cells from (c). $N = 2$ biologically independent experiments, n > 50 cells pwe experiment. Data are shown as mean ± SD. Statistical significance was determined using a 2-way ANOVA test**.** (Šídák multiple comparison test). *P*-values are indicated on the graphs. Source data are provided in the Source Data file.

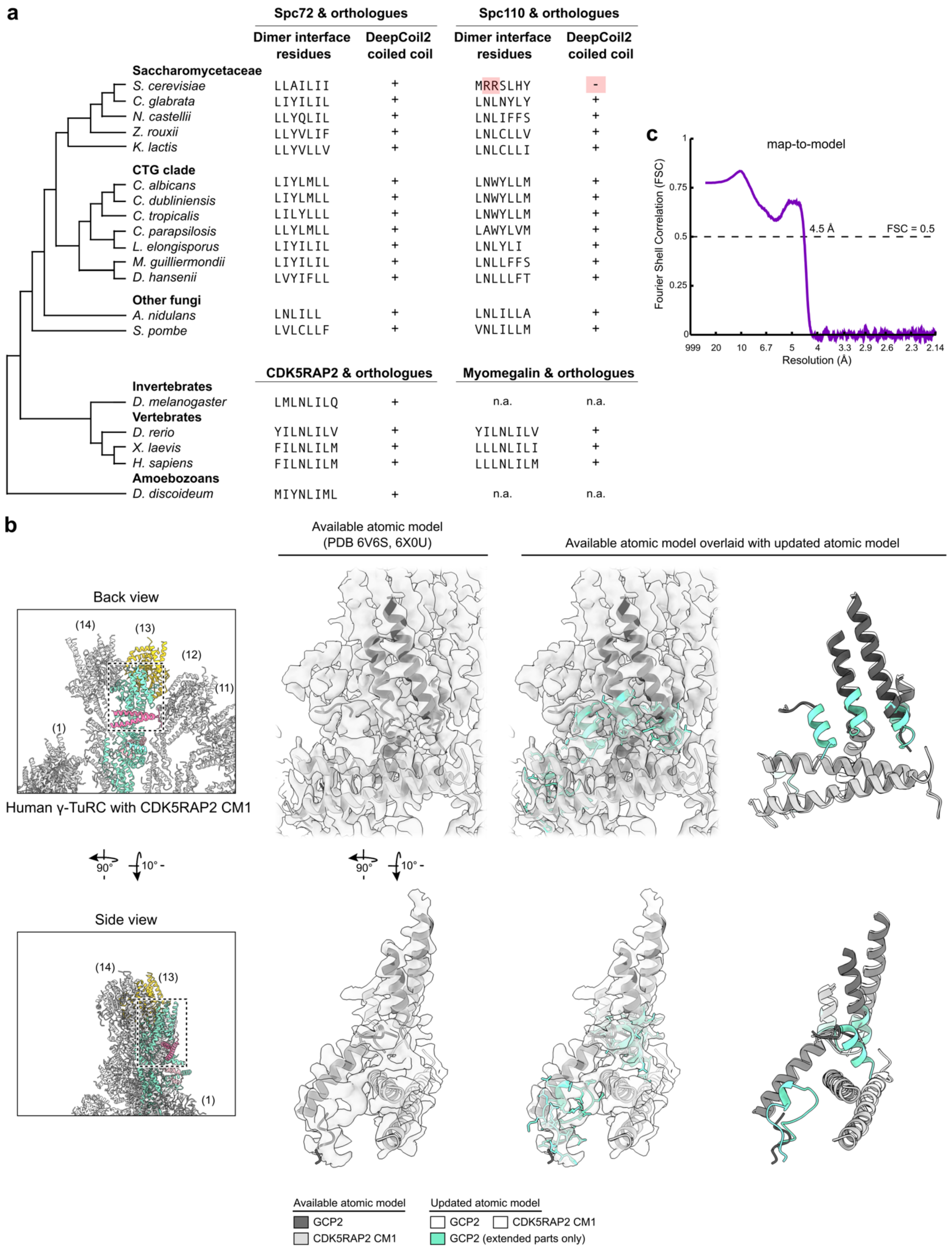
a
Spc72 & orthologues
Spc110 & orthologues
Dimer interface residues
DeepCoil2 coiled coil
Saccharomycetaceae
S. cerevisiae LLAILII + MRRSLHY -
C. glabrata LIYILIL + LNLNYLY +
N. castellii LLYQLIL + LNLIFFS +
Z. rouxii LLYVLIF + LNLCLLV +
K. lactis LLYVLLV + LNLCLLI +
CTG clade
C. albicans LIYLMLL + LNWYLLM +
C. dubliniensis LIYLMLL + LNWYLLM +
C. tropicalis LILYLLL + LNWYLLM +
C. parapsilosis LLYLMLL + LAWYLVM +
L. elongisporus LIYILIL + LNLYLI +
M. guilliermondii LIYILIL + LNLLFFS +
D. hansenii LVYIFLL + LNLLLFT +
Other fungi
A. nidulans LNLILL + LNLILLA +
S. pombe LVLCLLF + VNLILLM +
CDK5RAP2 & orthologues
Myomegalin & orthologues
Invertebrates
D. melanogaster LMLNLILQ + n.a. n.a.
Vertebrates
D. rerio YILNLILV + YILNLILV +
X. laevis FILNLILM + LLLNLILI +
H. sapiens FILNLILM + LLLNLILM +
Amoebozoans
D. discoideum MIYNLIML + n.a. n.a.
b
Back view
(14)
(13)
(12)
(11)
(1)
Human γ-TuRC with CDK5RAP2 CM1
Side view
90°
10°
Available atomic model (PDB 6V6S, 6X0U)
Available atomic model overlaid with updated atomic model
Available atomic model
GCP2
CDK5RAP2 CM1
Updated atomic model
GCP2
CDK5RAP2 CM1
GCP2 (extended parts only)
c
map-to-model
Fourier Shell Correlation (FSC)
1
0.75
0.5
0.25
0
4.5 Å
FSC = 0.5
999 20 10 6.7 5 4 3.3 2.9 2.6 2.3 2.14
Resolution (Å)

Supplementary Figure 8. Evolutionary conservation of CM1 motif dimerisation and interaction elements.

(a) Predicted dimerisation of CM1 motifs in orthologues of Spc72 and Spc110 in fungi, as well as orthologues of CDK5RAP2 and myomegalin in other eukaryotes along the evolutionary tree. Plus (+) and minus (-) indicate predicted dimerisation by DeepCoil2; residues predicted to be at the intra-coiled-coil dimer interface are shown. Red boxes indicate residues incompatible with coiled-coil formation. **(b)** Comparison of the available atomic model for the CDK5RAP2 CM1 motif bound to the human γ-TuRC (composite of PDB 6V6S[7] and 6X0V[8], shown in light and dark grey) and the updated atomic model, in which missing interaction elements present in the cryo-EM reconstruction (EMD 21985) have been built (green). In the bottom panels, only density segments corresponding to CM1 and interaction elements on GCP2 are shown to aid visual interpretation. **(c)** Map-to-model Fourier shell correlation (FSC) of the updated atomic model for the binding of the CDK5RAP2 CM1 motif to the human γ-TuRC. The resolution value at which the FSC crosses the FSC=0.5 threshold is indicated.

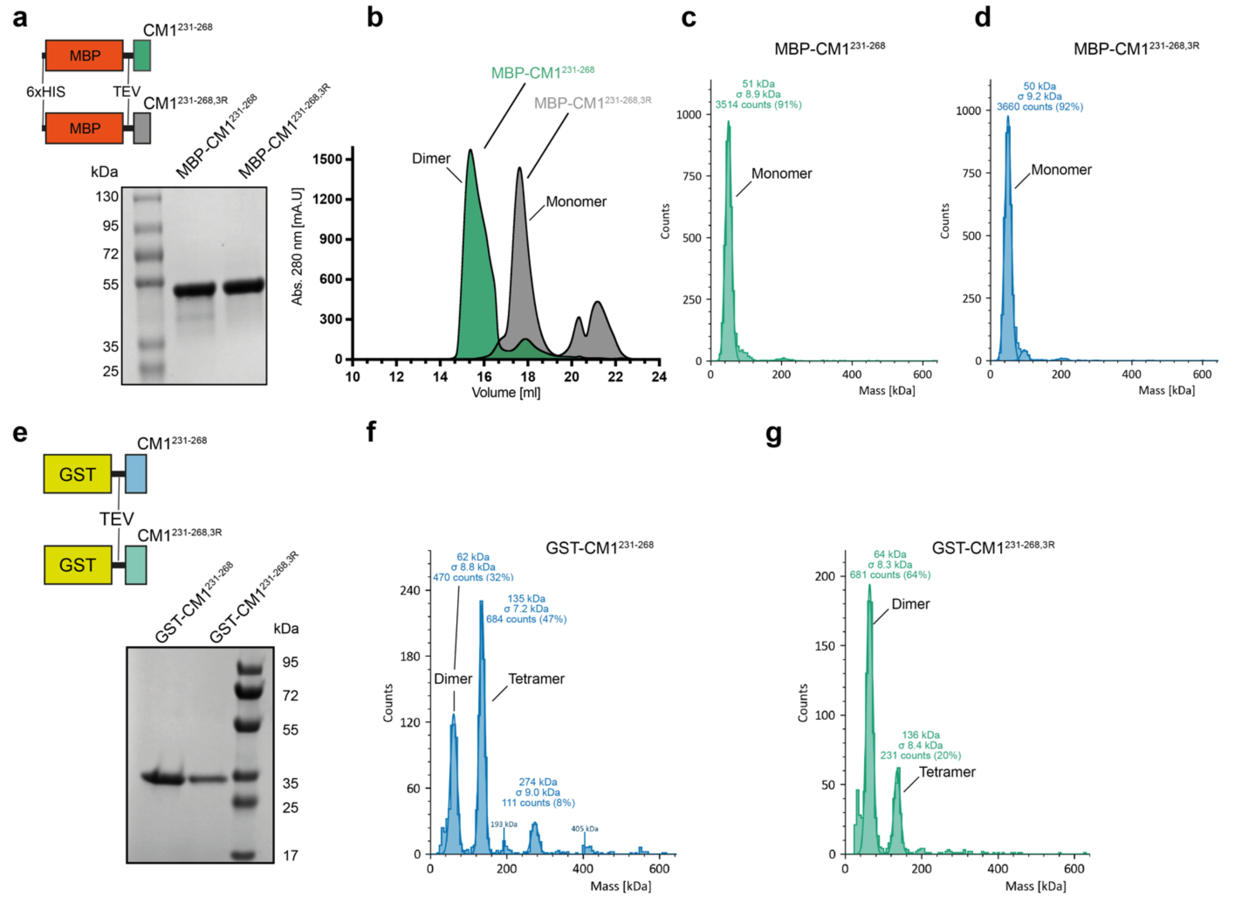

Supplementary Figure 9. The CM1 3R mutant disrupts dimerisation of the isolated CM1 motif to a large extent.

(a) Top: Constructs for *E. coli* expression of His-MBP-tagged Spc72 CM1 motif variants (MBP-CM1$^{231\text{-}268}$ and MBP-CM1$^{231\text{-}268,3R}$). Bottom: Coomassie-stained SDS-PAGE gel of purified proteins. **(b)** SEC chromatogram of MBP-CM1$^{231\text{-}268}$ (WT, 100 µM; green) and MBP-CM1$^{231\text{-}268,3R}$ (3R, 100 µM; gray) after His affinity purification. $N = 3$ biologically independent experiments. **(c,d)** Mass photometry analysis of MBP-CM1$^{231\text{-}268}$ (c) and MBP-CM1$^{231\text{-}268,3R}$ (d). Protein concentration during mass photometry experiments was < 40 nM. $N = 2$ biologically independent experiments. **(e)** Top: *E. coli* expression constructs of GST-tagged Spc72 CM1 motif constructs (GST-CM1$^{231\text{-}268}$ and GST-CM1$^{231\text{-}268,3R}$). Bottom: Coomassie-stained SDS-PAGE gel of purified proteins. $N = 2$ biologically independent experiments. **(f,g)** Mass photometry analysis of GST-CM1$^{231\text{-}268}$ (f) and GST-CM1$^{231\text{-}268,3R}$ (g). Protein concentration in mass photometry experiments was < 40 nM in both cases. Tetramer formation suggests CM1-mediated dimerisation of two GST-mediated dimers. $N = 1$ biologically independent experiment. Source data are provided in the Source Data file.

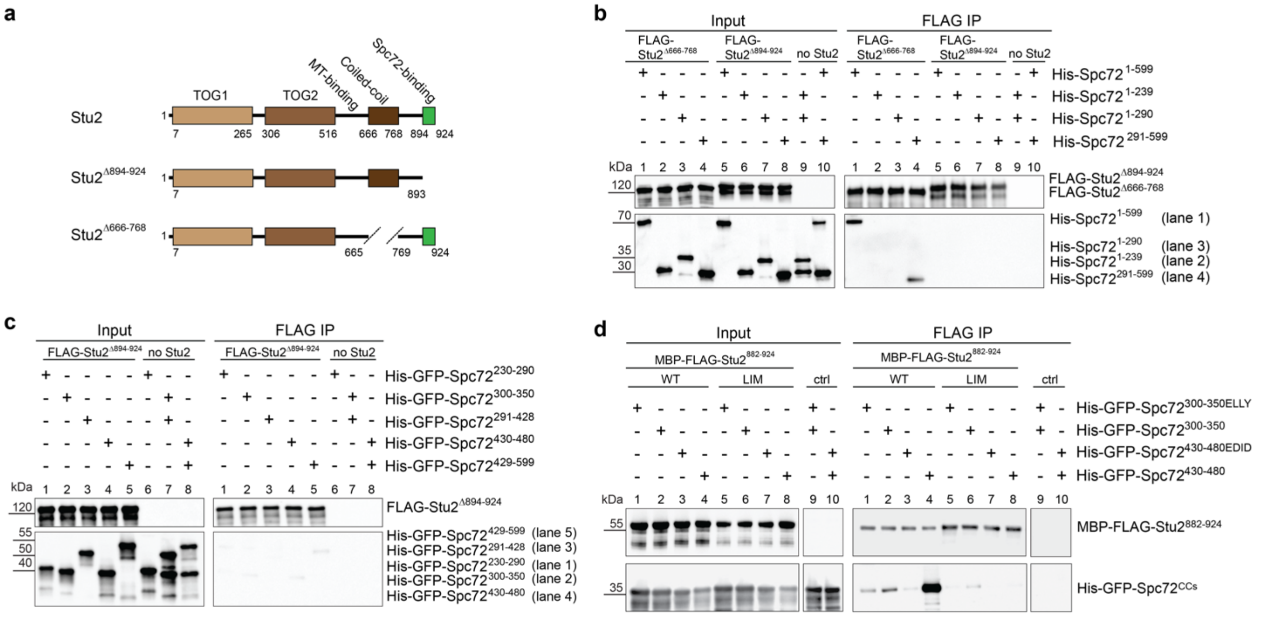

Supplementary Figure 10. Structure-guided interaction analysis of Stu2 and Spc72.

(a) Scheme highlighting different functional domains of Stu2 (left), based on *S. cerevisiae* Stu2 [1], as well as Stu2 constructs used for immunoprecipitation experiments in panels (b) and (c). **(b)** Anti-FLAG immunoprecipitation experiments with Stu2 constructs outlined in panel (a) (lanes 1-8) or without FLAG-Stu2 (lanes 9,10) and different His-Spc72 fragments (outlined in Fig. 6a), demonstrating that the C-terminal helix but not the coiled-coil region of Stu2 is required for interaction with Spc72. Experiment shown in panel (b) is from the same pull-down experiment and blot shown in **Fig. 6a.** $\boldsymbol{N = 2}$ biologically independent experiments. (**c**) Guided by AlphaFold2 predictions (shown in Fig. 6c), anti-FLAG immunoprecipitation experiments were used to confirm that the primary and secondary binding sites (confirmed in Fig. 6b) on the two coiled-coil modules of Spc72 (outlined in Fig. 6b) interact with the C-terminal helix of Stu2. The experiment shown in panel (c) is from the same pull-down experiment and blot shown in Fig. 6b. $N = 2$ biologically independent experiments. Corresponding input and FLAG IP lanes were labeled with the same number to aid comparison. **(d)** Anti-FLAG immunoprecipitation experiments with wild-type MBP-FLAG-Stu$^{882\text{-}924}$ (lanes 1-4), the MBP-FLAG-Stu2$^{882\text{-}924LIM}$ (L906A, I910A, M913A) mutant (lanes 5-8) or without MBP-FLAG-Stu2 (lanes 9,10; ctrl) and His-GFP-Spc72$^{300\text{-}350}$, the His-GFP-Spc72$^{300\text{-}350ELLY}$ (E317R, L319A, L321R, Y326A) mutant, His-GFP-Spc72$^{430\text{-}480}$ and the His-GFP-Spc72$^{430\text{-}480EDID}$ (E455A, D456A, I458A, D462A) mutant, demonstrating that site-directed mutations affect the binding between the Stu2 C-terminal helix and the coiled-coil modules of Spc72. Anti-FLAG and anti-His antibodies were used for visualisation of protein components in panels (b-d). $N = 2$ biologically independent experiments. Source data are provided in the Source Data file.

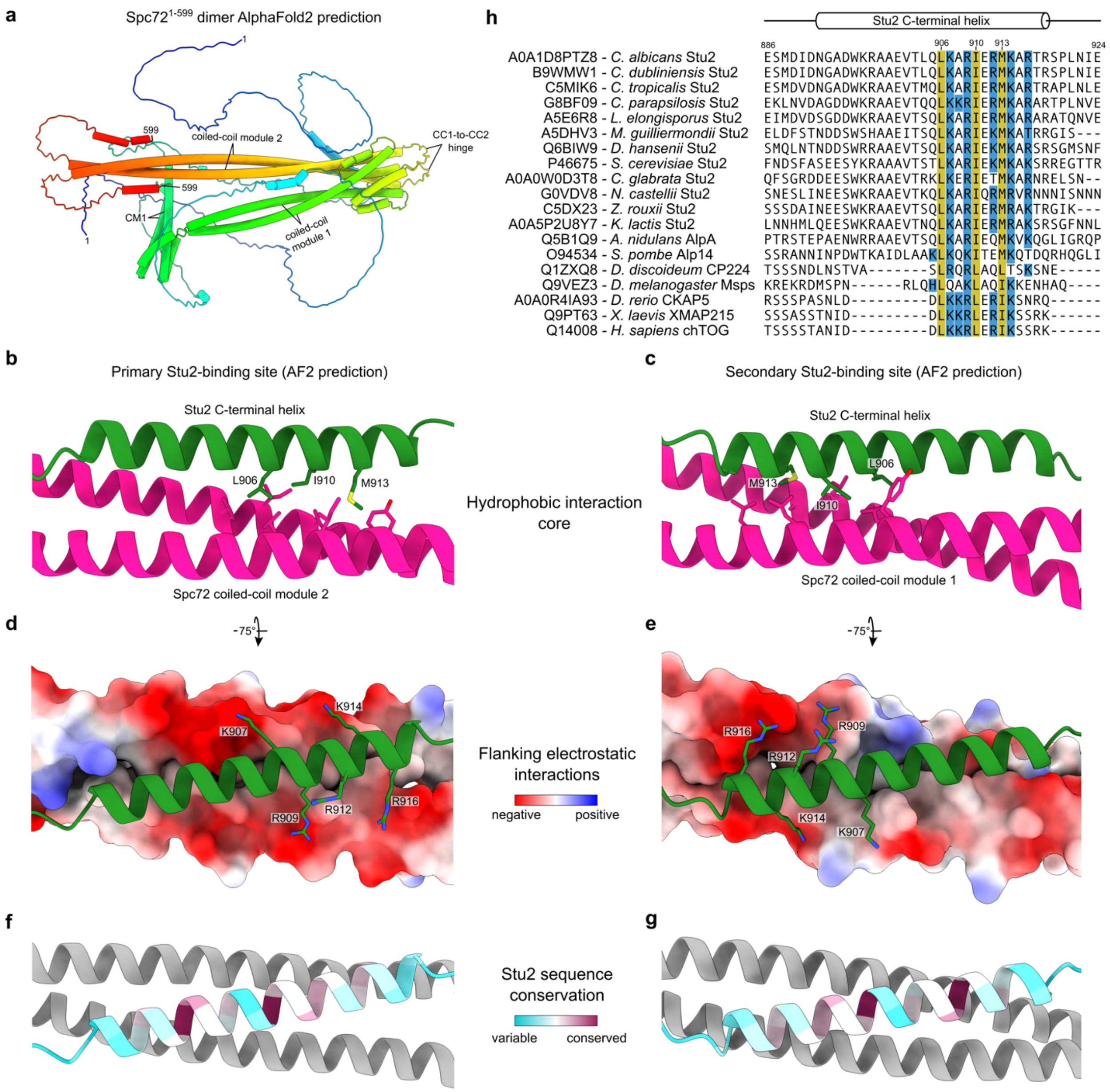

Supplementary Figure 11. AlphaFold2 predicts two binding sites of the Stu2 C-terminal helix on Spc72$^{1\text{-}599}$ identified through immunoprecipitations.

(a) Top-ranked AlphaFold2 model of dimeric Spc72$^{1\text{-}599}$, showing two coiled-coil modules following the dimeric CM1 motif, following a large, mostly unstructured stretch. CC1/CC2 = coiled-coil module 1/2. Chains are coloured from N- to C-terminus using a rainbow ramp from blue to red; N- and C-terminus (residue 1 and 599, respectively) are indicated. **(b,c)** Visualisation of the hydrophobic core interaction on the AlphaFold2-predicted binding interfaces of the primary (b) and secondary (c) binding sites of Stu2 (green) on Spc72 (pink) coiled-coil modules 2 and 1, respectively. Conserved Stu2 residues are numbered. **(d,e)** Predicted electrostatic interactions with positively charged Stu2 (green) residues on a negatively charged surface of Spc72 (shown with electrostatic surface colouring) on the AlphaFold2-predicted binding interfaces of the primary (d) and secondary (e) binding sites. Conserved positively

charged residues of Stu2 are numbered. **(f,g)** Stu2 sequence conservation mapped onto the AlphaFold2-predicted binding interfaces of the primary (f) and secondary (g) binding sites on Spc72 (shown in grey), showing that conserved residues mainly face Spc72, while variable residues mainly point away from the predicted interfaces. **(h)** Sequence conservation of the C-terminal helix of Stu2 involved in binding Spc72 in Stu2/CKAP5/XMAP215/chTOG homologues throughout fungi and other eukaryotes. Conserved hydrophobic residues involved in the AlphaFold2-predicted interface (see panels b,c) are highlighted in yellow, whereas positive charges are highlighted in blue. UniProt IDs are indicated.

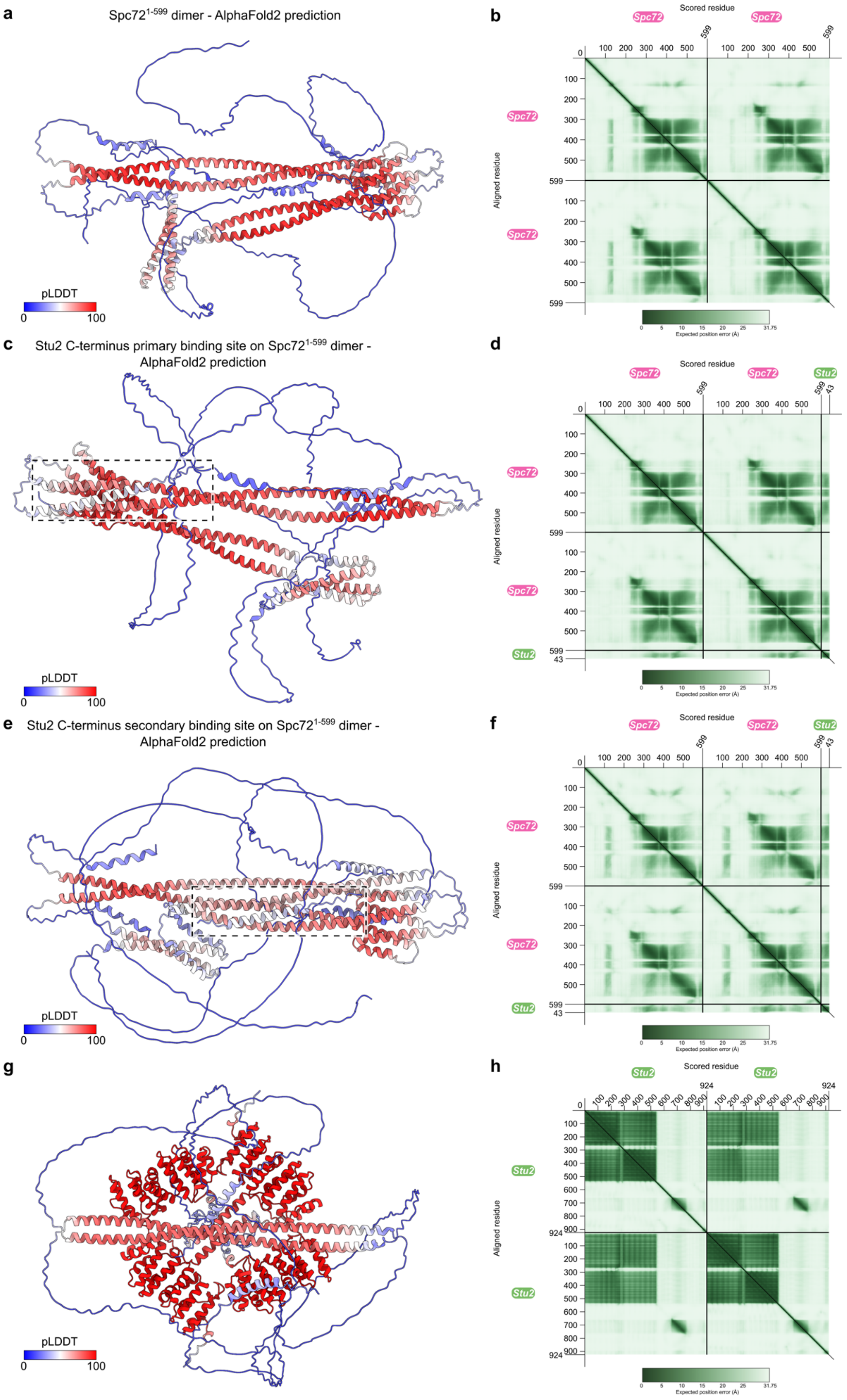
a
Spc72 1-599 dimer - AlphaFold2 prediction
pLDDT
0
100
b
Scored residue
Spc72
Spc72
Aligned residue
Expected position error (Å)
c
Stu2 C-terminus primary binding site on Spc72 1-599 dimer - AlphaFold2 prediction
pLDDT
0
100
d
Scored residue
Spc72
Spc72
Stu2
Aligned residue
Expected position error (Å)
e
Stu2 C-terminus secondary binding site on Spc72 1-599 dimer - AlphaFold2 prediction
pLDDT
0
100
f
Scored residue
Spc72
Spc72
Stu2
Aligned residue
Expected position error (Å)
g
pLDDT
0
100
h
Scored residue
Stu2
Stu2
Aligned residue
Expected position error (Å)

Supplementary Figure 12. pLDDT and PAE matrices for AlphaFold2 predictions.

(a,b) Top-ranked AlphaFold2 prediction of dimeric *C. albicans* $Spc72^{1-599}$ coloured by pLDDT (predicted local-distance difference test) (a) and associated PAE (predicted aligned error) matrix (b). **(c,d)** Top-ranked AlphaFold2 prediction for the interaction between dimeric *C. albicans* $Spc72^{1-599}$ and the 43 C-terminal residues of *C. albicans* Stu2 at the primary binding site, coloured by pLDDT (c), and associated PAE matrix (d). Dashed box indicates Stu2 binding site. **(e,f)** Top-ranked AlphaFold2 prediction for the interaction between dimeric *C. albicans* $Spc72^{1-599}$ and the 43 C-terminal residues of *C. albicans* Stu2 at the secondary binding site, coloured by pLDDT (e), and associated PAE matrix (f). Dashed box indicates Stu2 binding site. **(g,h)** Top-ranked AlphaFold2 prediction of the dimeric full-length *C. albicans* Stu2, coloured by pLDDT (g), and associated PAE matrix (h). Colour scales as indicated. PAE matrices were visualised using PAE viewer[9].

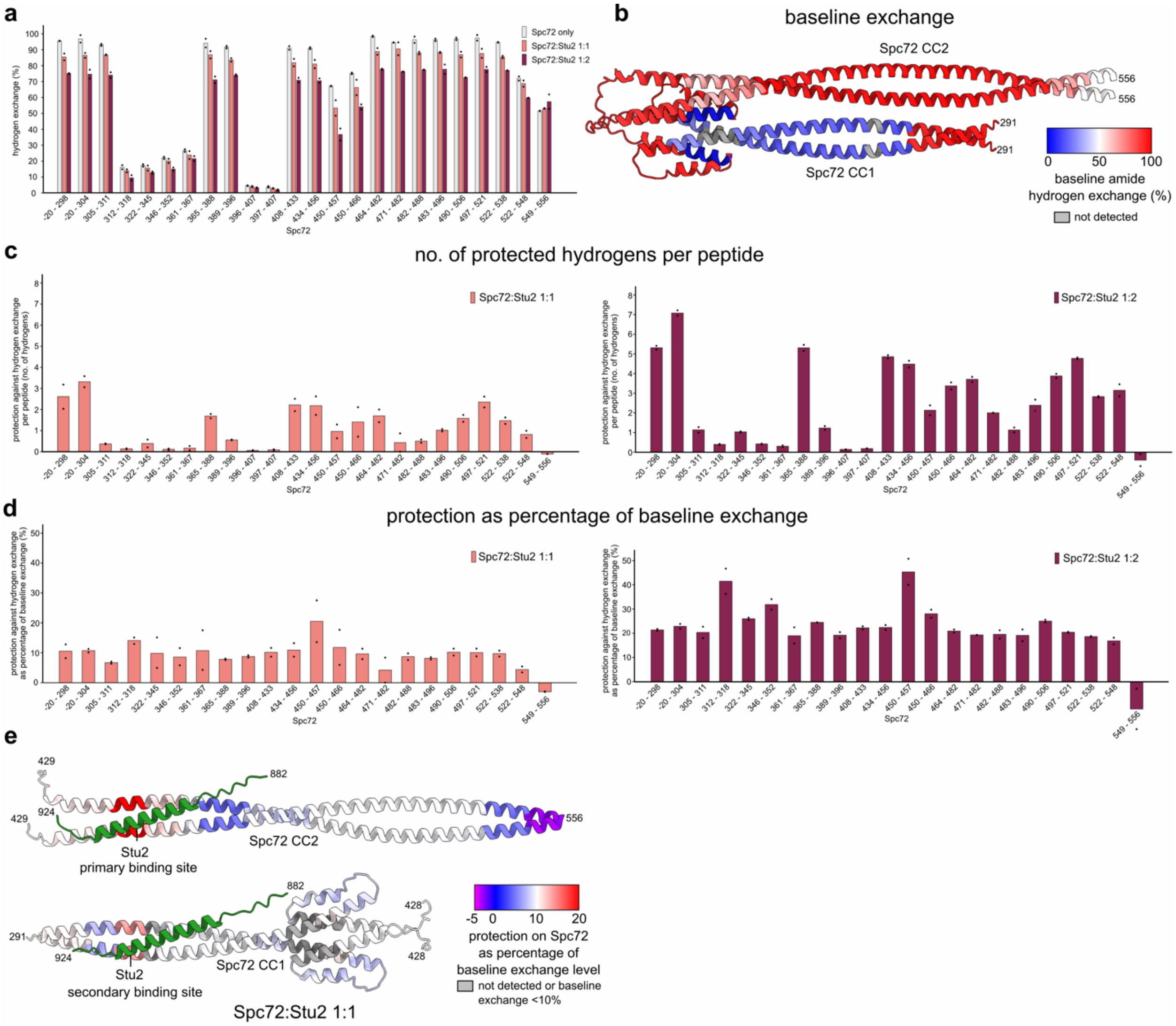

Supplementary Figure 13. Hydrogen exchange mass spectrometry of Spc72$^{291\text{-}599}$ in the presence or absence of Stu2.

(a) Percent exchange of His-Spc72$^{291\text{-}599}$ backbone amide protons for deuterons after 30 seconds exposure in deuterated buffer in the absence (light grey), or presence of full-length FLAG-Stu2 in a 1:1 (light red) or 1:2 (burgundy red) His-Spc72$^{291\text{-}599}$:FLAG-Stu2 molar ratio. **(b)** Baseline exchange (i.e. in the absence of FLAG-Stu2) of His-Spc72$^{291\text{-}599}$ visualised on the AlphaFold2 prediction of Spc72 (see Supplementary Fig. 12c,d). Colour scale as indicated. **(c)** Hydrogen exchange of His-Spc72$^{291\text{-}599}$ in the presence of FLAG-Stu2 in 1:1 (left) or 1:2 (right) His-Spc72$^{291\text{-}599}$:FLAG-Stu2 molar ratio as number of protected hydrogens per peptide, i.e., not corrected for peptide length or baseline exchange. **(d)** As in (c), but corrected for the baseline exchange in the absence of FLAG-Stu2, which is the maximum exchange that can theoretically be protected by addition of FLAG-Stu2. **(e)** Visualisation of baseline exchange-corrected protection on the AlphaFold2 prediction of Spc72 at 1:1 His-Spc72$^{291\text{-}599}$:FLAG-Stu2 molar ratio. The corresponding visualisation for 1:2 His-Spc72$^{291\text{-}599}$:FLAG-Stu2 molar ratio is shown in Fig. 6d. Data

in (a), (c) and (d) are shown per detected peptide; residue numbers are indicated. Negative numbers indicate residues in the purification tag. Individual data points are indicated as dots.

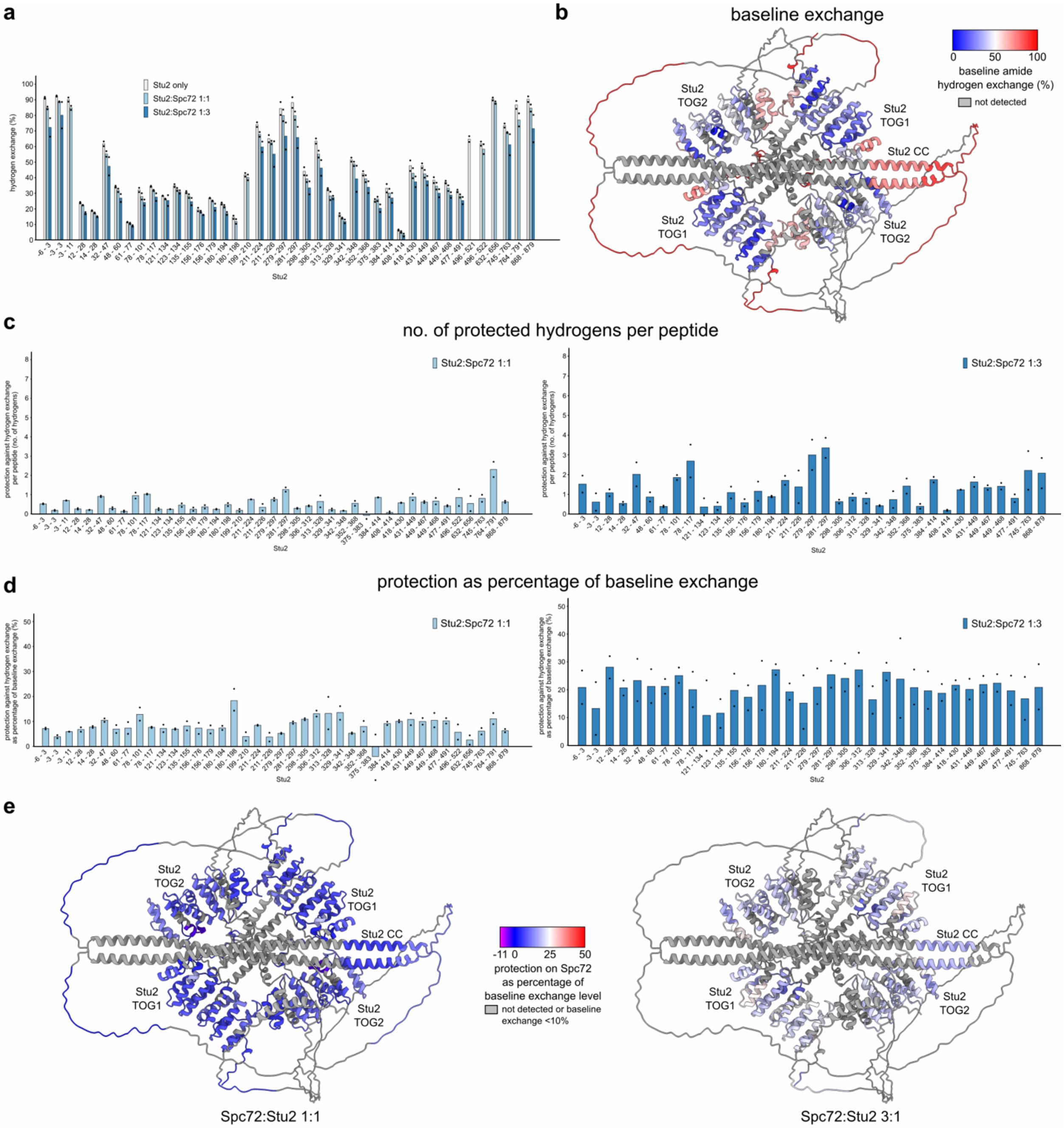

Supplementary Figure 14. Hydrogen exchange mass spectrometry of Stu2 in the presence or absence of Spc72$^{291\text{-}599}$.

(a) Percent exchange of FLAG-Stu2 backbone amide protons for deuterons after 30 seconds exposure in deuterated buffer in the absence (light grey), or presence of His-Spc72 in a 1:1 (light blue) or 3:1 (dark blue) His-Spc72$^{291\text{-}599}$:FLAG-Stu2 molar ratio. **(b)** Baseline exchange (i.e. in the absence of His-Spc72$^{291\text{-}599}$) of FLAG-Stu2 visualised on the AlphaFold2 prediction of Stu2 (see Supplementary Fig. 12g,h). Colour scale as indicated. **(c)** Hydrogen exchange of FLAG-Stu2 in the presence of His-Spc72$^{291\text{-}599}$ in 1:1 (left) or 3:1 (right) His-Spc72$^{291\text{-}599}$:FLAG-Stu2 molar ratio as number of protected hydrogens per peptide, i.e., not corrected for peptide length or baseline exchange.

(d) As in (c), but corrected for the baseline exchange in the absence of His-Spc72$^{291-599}$, which is the maximum exchange that can theoretically be protected by addition of His-Spc72$^{291-599}$. **(e)** Visualisation of baseline exchange-corrected protection on the AlphaFold2 prediction of FLAG-Stu2 at 1:1 His-Spc72$^{291-599}$:FLAG-Stu2 (left) or 3:1 His-Spc72$^{291-599}$:FLAG-Stu2 (right) molar ratio. Data in (a), (c) and (d) are shown per detected peptide; residue numbers are indicated. Negative numbers indicate residues in the purification tag. Individual data points are indicated as dots.

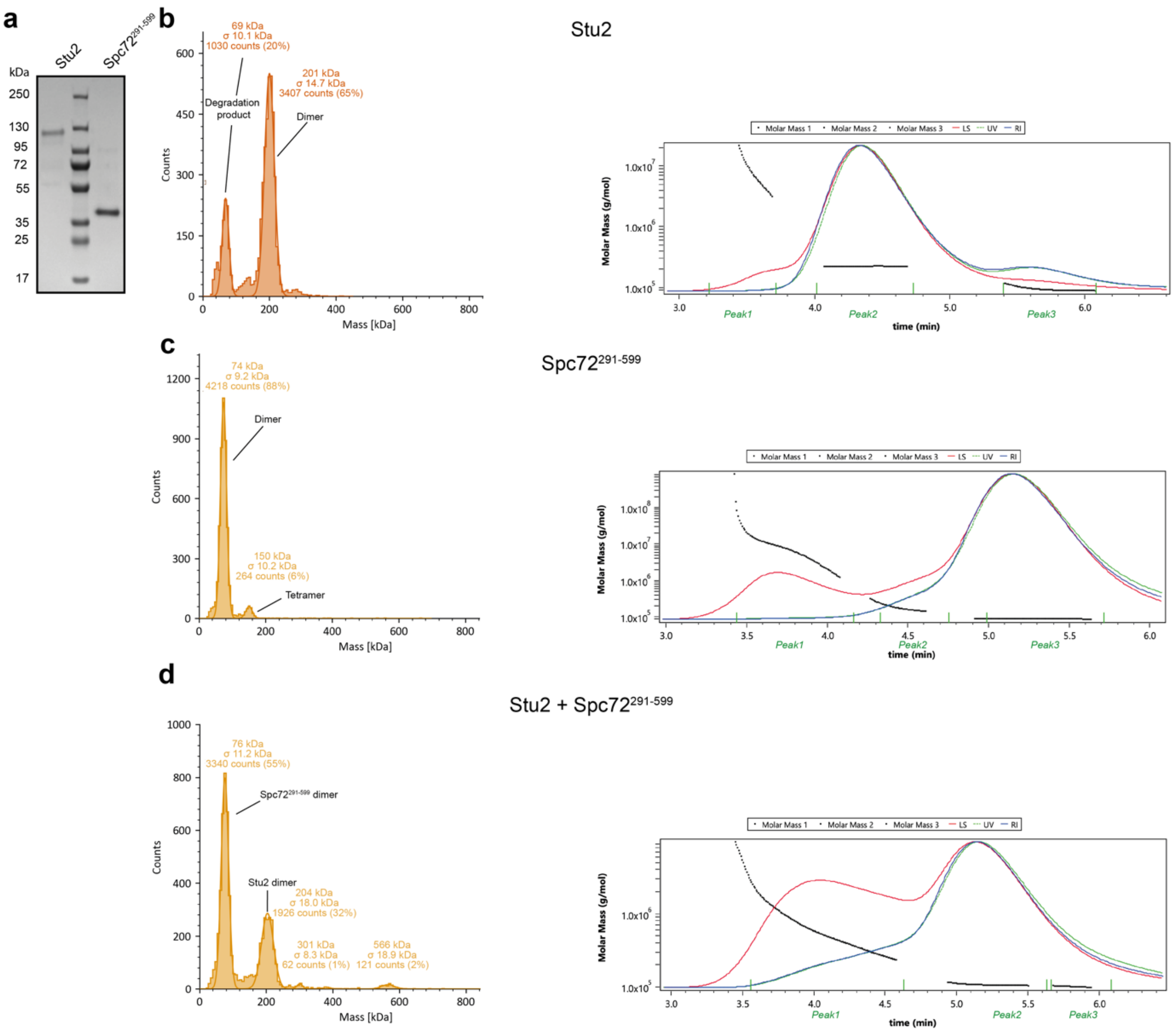

e

	Peak 1			Peak 2			Peak 3		
	Spc72291-599	Stu2	Stu2 : Spc72291-599 1:6	Spc72291-599	Stu2	Stu2 : Spc72291-599 1:6	Spc72291-599	Stu2	Stu2 : Spc72291-599 1:6
**Mw (kDa)**	4971.0	5645.2	479.0	173.3	219.5	106.6	88.3	94.7	101.0
**Uncertainty**	0.4 %	1.8 %	1.6 %	1.5 %	0.5 %	0.6 %	0.6 %	2.8 %	0.1 %
**Polydispersity (Mw/Mn)**	1.818	1.197	1.286	1.042	1.000	1.001	1.000	1.007	1.000
**Uncertainty**	0.567 %	2.292 %	2.316 %	2.055 %	0.743 %	0.860 %	0.791 %	3.989 %	0.172 %
**Mass fraction (%)**	0.5	0.2	21.4	6.6	85.2	67.2	92.9	14.6	11.4

Supplementary Figure 15. Stoichiometry of Spc72 and Stu2.

(**a**) Coomassie-stained SDS-PAGE analysis of His-Spc72$^{291\text{-}599}$ and FLAG-Stu2 purifications. *N* = 3 biologically independent experiments. (**b**) Left: Mass photometry analysis of FLAG-Stu2. Right: SEC-MALS experiment of FLAG-Stu2. Run data are summarised in (e). (**c**) Left: Mass photometry analysis of His-Spc72$^{291\text{-}599}$. Right: SEC-MALS experiment of His-Spc72$^{291\text{-}599}$. Run data are summarised in (e). (**d**) Left: Mass photometry analysis of His-Spc72$^{291\text{-}599}$ mixed with FLAG-Stu2 (ratio 2:1). Right: SEC-MALS experiment of His-Spc72$^{291\text{-}599}$ mixed with FLAG-Stu2 (6:1 molar ratio). (**e**) Table summarising SEC-MALS analysis. Protein concentration was in the nM range in mass-photometry experiments and in the µM range in SEC-MALS experiments. Mass photometry was performed in

$N = 2$ technical replicates. Mass photometry and SEC-MALS were performed in $N = 1$ biologically independent experiment. Source data are provided in the Source Data file.

Supplementary Table 1. Cryo-EM data collection, refinement and validation statistics.

	Candida albicans γ-TuSC in complex with Spc72$^{1-599}$ within ring-like higher oligomer (EMDB-51971) (PDB 9H9Q)	Full ring of *Candida albicans* γ-TuSC in complex with Spc72$^{1-599}$ (EMDB-51972) (PDB 9H9R)	Spokes 12 and 13 of the human γ-TuRC in complex with CDK5RAP2 (PDB 9H9P)
**Data collection and processing**			Obtained from EMDB-21985
Magnification	81000 x	81000 x	
Voltage (kV)	300	300	
Electron exposure (e–/Å^2)	47	47	
Defocus range (μm)	-1 to -2.6	-1 to -2.6	
Pixel size (Å)	1.07	1.07	
Symmetry imposed	C1	C1	
Initial particle images (no.)	1711419	1711419	
Final particle images (no.)	129908	8261	
Map resolution (Å)	3.6	8.2	
FSC threshold	0.143	0.143	
Map resolution range (Å)	3.4-5.5	5.7-23.8	
**Refinement**			
Initial model used (PDB code)	*de novo*	xxxx (model in left column)	6V6S and 6X0V
Model resolution (Å)	3.7	8.3	4.5
FSC threshold	0.5	0.5	0.5
Model resolution range (Å)			
Map sharpening *B* factor (Å^2)	Local resolution filtering, -74	Local resolution filtering, -168	No additional sharpening on top of EMDB-21985
Model composition			
Non-hydrogen atoms	36618	128163	16352
Protein residues	4486	15701	2016
Ligands	0	0	2
B factors (Å^2)			
Protein	69.85	69.85	40.87
Ligand			
R.m.s. deviations			
Bond lengths (Å)	0.005	0.005	0.002
Bond angles (°)	1.008	1.007	0.529
Validation			
MolProbity score	1.23	1.25	1.47
Clashscore	4	4	6.21
Poor rotamers (%)	0.6	0.6	0.2
Ramachandran plot			
Favored (%)	98	98	97
Allowed (%)	2	2	3
Disallowed (%)	0	0	0

Supplementary Table 2. Primer list.

Primer Name	Sequence (5' to 3')	Source	Identifier
pFastbac1-FLAG-Stu2_Fwd	CCGTCCCACCATCGGGCGCGGATCCATGGATTACAAAGATGATGATGATAAGCTGGAAGTTCTGTTCCAGGGGCCCATGAGCACTGAAGAAGAAG	this study	N/A
pFastbac1-Stu2_Rev	TTCCGCGCGCTTCGGACCGGGATCTCATTCTATGTTCAGTGGAC	this study	N/A
pIDC-FLAG-Stu2_Fwd	TAAAAAAACCTATAAATATGGATTACAAAGATGATGATG	this study	N/A
pIDC-FLAG-Stu2_Rev	TCGAGACTGCAGGCTCTAGATCATTCTATGTTCAGTGGA	this study	N/A
pET28b-Spc72_Fwd	CTGGTGCCGCGCGGCAGCCATATGTCGAACTTAAGTATCAATG	this study	N/A
Spc72-Thr426_Rev	AGTAAAGAGGTCGTGTGGTTTATTATTGTACTGATG	this study	N/A
Spc72-Arg410_Fwd	CGTCTGGATCAGTCACATCAGTACAATAATAAACCAC	this study	N/A
pET28b-Spc72_Rev	AGTGGTGGTGGTGGTGGTGCTCGAGTCAGTTATTGTCGTTCGTGGTC	this study	N/A
pIDS-Spc72$^{1-599}$_Fwd	AAAACCTATAAATATGCATCATCATCATCAT	this study	N/A
pIDS-Spc72$^{1-599}$_Rev	GACTGCAGGCTCTAGACTAGTTGAGCTCATTG	this study	N/A
MultiBac_vector_fwd	TCTAGAGCCTGCAGTCTCG	Würtz et al., 2021[10]	N/A
MultiBac_vector_rev	ATATTTATAGGTTTTTTTATTACAAAACTG	Würtz et al., 2021[10]	N/A
pACEBac1-His-Spc97_Fwd	AAAACCTATAAATATGTCGTACTACCATCA	this study	N/A
pACEBac1-His-spc97_Rev	ACTGCAGGCTCTAGACTATTCTGGAAAGCA	this study	N/A
pIDC-Spc98_Rev	GACTGCAGGCTCTAGACTACAGTAATTTACTC	this study	N/A
pIDK-Tub4-Fwd	AAAACCTATAAATATGCCAGGTGAAACA	this study	N/A

pIDK-Tub4-Rev	ACTGCAGGCTCTAGACTAAATACCCATATC	this study	N/A
Combination_vector_fwd	TTCGCGACCTACTCCGGA	Würtz et al., 2021[10]	N/A
Combination_vector_rev	CAGATAACTTCGTATAATGTATGCT	Würtz et al., 2021[10]	N/A
Combination_insert_fwd	ATACGAAGTTATCTGTTCGCGACCTACTCCGGA	Würtz et al., 2021[10]	N/A
Combination_insert_rev	GGAGTAGGTCGCGAAGATCCAGACATGATAAGATACATTG	Würtz et al., 2021[10]	N/A
pACEBac1-FLAG-Spc97_Fwd	TAAAAAAACCTATAAATATGGATTACAAAGATGATGATGATAAGCTGGAAGTTCTGTTCCAGGGGCCCATGAATACGTTCTCCTCTC	this study	N/A
pACEBac1-FLAG-Spc97_Rev	TCGAGACTGCAGGCTCTAGACTATTCTGGAAAGCACAATTC	this study	N/A
pIDC-Spc98_Fwd	TAAAAAAACCTATAAATATGGCTCTGAACAAAGTG	this study	N/A
pIDC-FLAG-Spc98_Fwd	TAAAAAAACCTATAAATATGGATTACAAAGATGATGATGATAAGCTGGAAGTTCTGTTCCAGGGGCCCATGGCTCTGAACAAAGTG	this study	N/A
pFastbac1-FLAG-Spc98_Fwd	TCCCACCATCGGGCGCGGATCCATGGATTACAAAGATGAT	this study	N/A
pIDC-GFP-Mzt1_Fwd	TAAAAAAACCTATAAATATGGTGAGCAAGGGCGAGGA	this study	N/A
pIDC-GFP-Mzt1_Rev	AGACTGCAGGCTCTAGATCAGTGGTGGTGGTGGT	this study	N/A
pIDS-Spc72$^{1\text{-}599:PA}$_Fwd	TAAAAAAACCTATAAATATGCACCACCACCACCACCATTC	this study	N/A
pIDS-Spc72$^{1\text{-}599:PA}$_F1	ACAAAAGAATCGTCAACTACAAAATCCAACTTAAATTAATG	this study	N/A
pIDS-Spc72$^{1\text{-}599:PA}$_R1	CATTAATTTAAGTTGGATTTTGTAGTTGACGATTCTTTTGT	this study	N/A
pIDS-Spc72$^{1\text{-}599:PA}$_Rev	TGTCGAGACTGCAGGCTCTAGACTAGTTGAGCTCATTGAGGGA	this study	N/A
pIDS-Spc72$^{1\text{-}599:3R}$_F1	TGATAGATCGTAATAACCTAGACCCGCATGAGTTTCACAC	this study	N/A
pIDS-Spc72$^{1\text{-}599:3R}$_R1	GTGTGAAACTCATGCGGGTCTAGGTTATTACGATCTATCA	this study	N/A

pFastbac1-FLAG-Stu2_F1	GTCCCACCATCGGGCGCGGATCCATGGATTA	this study	N/A
pFastbac1-FLAG-Stu2$^{\Delta 894-924}$_Fwd	GTCCCACCATCGGGCGCGGATCCATGGATTA	this study	N/A
pFastbac1-FLAG-Stu2$^{\Delta 894-924}$_Rev	CGCTTCGGACCGGGATCcTCATCCATTGTCAATGTCCAT	this study	N/A
pFastbac1-FLAG-Stu2$^{\Delta 666-768}$_Fwd	AGCTCAGTCACTAATGAAGAAGTTAACATCAGTTCCAGCAATTCCA	this study	N/A
pFastbac1-FLAG-Stu2$^{\Delta 666-768}$_Rev	TGGAATTGCTGGAACTGATGTTAACTTCTTCATTAGTGACTGAGCT	this study	N/A
pET28b-Spc72$^{1-599}$_Rev	AGTGGTGGTGGTGGTGGTGCTCGAGTCAGTTGAGCTCATTGAGGGAAG	this study	N/A
pET28b-Spc72$^{1-239}$_Rev	TGGTGGTGGTGGTGCTCGAGTCAACGCGATTTAGTCGTG	this study	N/A
pET28b-Spc72$^{1-290}$_Rev	TGGTGGTGGTGGTGCTCGAGTCACTCGTTGTTCATGATGT	this study	N/A
pET28b-Spc72$^{291-599}$_Rev	TGCCGCGCGGCAGCCATATGAATAATCCGCTGTCGAC	this study	N/A
pRS315_Fwd	TAGTTATTTGCGGTGACTCGAG	this study	N/A
pRS315_Rev	CATGGATCCACTAGTTCTAGA	this study	N/A
ScSpc72_F1	GCTCTAGAACTAGTGGATCCA	this study	N/A
ScSpc72_R1	CTCGAGTCACCGCAAATAACTA	this study	N/A
ScSpc72^{3R}_F2	CAAGTACTCTATGAATACATTCGCAGAATC	this study	N/A
ScSpc72^{3R}_R2	GATTCTGCGAATGTATTCATAGAGTACTTG	this study	N/A
ScSpc72$^{\Delta P55-N62}$_F2	TCATAACGATCCCATCAAGAACAAAGTCAAAAATTTGGA	this study	N/A
ScSpc$^{\Delta P55-N62}$_R2	TCCAAATTTTTGACTTTGTTCTTGATGGGATCGTTATGA	this study	N/A
pET28b-GFP-Fwd	CGGGATCCGAATTCGAGCT	this study	N/A
pET28b-GFP-Rev	CCCCTGGAACAGAACTTC	this study	N/A
pET28b-GFP-Spc72$^{230-290}$_Fwd	GAAGTTCTGTTCCAGGGGCCAATGAACAATATCGCGACCA	this study	N/A
pET28b-GFP-Spc72$^{230-290}$_Rev	AGCTCGAATTCGGATCCCGCTCGTTGTTCATGATGT	this study	N/A
pET28b-GFP-Spc72$^{300-350}$_Fwd	GAAGTTCTGTTCCAGGGGCCAATGCAAACATCTACTCTG	this study	N/A
pET28b-GFP-Spc72$^{300-350}$_Rev	CTCGAATTCGGATCCCGAATGCGGCTTTCGTAGT	this study	N/A

pET28b-GFP-Spc72$^{291-428}$_Fwd	GAAGTTCTGTTCCAGGGGCCAATGAATAATCCGCTGTCGA	this study	N/A
pET28b-GFP-Spc72$^{291-428}$_Rev	AGCTCGAATTCGGATCCCGTGGCGGAGTAAAGAGGT	this study	N/A
pET28b-GFP-Spc72$^{430-480}$_Fwd	GAAGTTCTGTTCCAGGGGCCAATGACCAGTAGCGAGTA	this study	N/A
pET28b-GFP-Spc72$^{430-480}$_Rev	AGCTCGAATTCGGATCCCGGTTTTGCAATTCATTTTTG	this study	N/A
pET28b-GFP-Spc72$^{429-599}$_Fwd	GAAGTTCTGTTCCAGGGGCCAATGTACACCAGTAGCGAGT	this study	N/A
pET28b-GFP-Spc72$^{429-599}$_Rev	CTCGAATTCGGATCCCGGTTGAGCTCATTGAGGGA	this study	N/A
Spc72$^{231-2683WT}$_fwd	ATCTTTATTTTCAGGGCGCCAATATCGCGACCACGACTAAATC	this study	N/A
Spc72$^{231-2683WT}$_Rev	TGCTCGAGTGCGGCCGCTTAGTTCCGGTCAATTAATTCCTG	this study	N/A
Spc72$^{231-268,3R}$ _Fwd	ATCTTTATTTTCAGGGCGCCAACATTGCTACTACTACGAAG	this study	N/A
Spc72$^{231-268,3R}$ _Rev	TGCTCGAGTGCGGCCGCTTAATTACGATCTATCAGCTCTTG	this study	N/A
Spc72-for-GST_Fwd	AATTCGAGCTCGAACAAC	this study	N/A
Spc72-for-GST _Rev	CATGGTATATCTCCTTCTTAAAG	this study	N/A
GST_insert_Fwd	AAGAAGGAGATATACCATGTCCCCTATACTAGGTTATTGGAAAATTAAG	this study	N/A
GST_insert_Rev	TTGTTGTTCGAGCTCGAATTTTTTGGAGGATGGTCGCC	this study	
petM41_MBP_PL_Fwd	TAAGCGGCCGCACTCGA	this study	N/A
petM41_MBP_PL_Rev	GGCGCCCTGAAAATAAAGATTCTC	this study	N/A
MBP_Rev	GGCGCCCTGAAAATAAAGA	this study	N/A
MBP-FLAG-Stu2$^{882-924}$_Fwd	TCTTTATTTTCAGGGCGCCGATTACAAAGATGATGA	this study	N/A
MBP-FLAG-Stu2$^{882-924}$_Rev	TCGAGTGCGGCCGCTTATCATTCTATGTTCAGTGGA	this study	N/A
S2-Spc72	TGAGTGTTACATTAAATATATTTATATATAAACGTATGATATTTAATCGATGAATTCGAGCTCG	this study	N/A
S3-Spc72	GAGTCATTGAGATCGAAACTTTTCAACCTATCAATCAACAATCCCCGTACGCTGCAGGTCGAC	this study	N/A
pMM5-Spc72_Fwd	TTTGAACGGGATCCccATGTCGAACTTAAGTA	this study	N/A

pMM5-Spc72_Rev	TACATGACTCGAGtcaTCAGTTATTGTCGTTC	this study	N/A
pMM6-Stu2_Fwd	GGGATCCCCCGGGctATGAGCACTGAAGAAGA	this study	N/A
pMM6-Stu2_Rev	ACGGTATCGATAAGCTTtcaTTCTATGTTCAGT	this study	N/A
Spc72$^{\Delta 300\text{-}350}$_Fwd	TCGACGTCATTAAGCAACCTGATTAATTAC	this study	N/A
Spc72$^{\Delta 300\text{-}350}$_Rev	GTAATTAATCAGGTTGCTTAATGACGTCGA	this study	N/A
Spc72$^{\Delta 430\text{-}480}$_Fwd	CTTTACTCCGCCATACCAGCTCGTGGAACAGA	this study	N/A
Spc72$^{\Delta 430\text{-}480}$_Rev	TCTGTTCCACGAGCTGGTATGGCGGAGTAAAG	this study	N/A
pRS316-GAL1-ScSpc72_Fwd	ATGTCTTTAATTAACAGTATGGTACGTCGATGGA	this study	N/A
pRS316-GAL1-ScSpc72_Rev	TGCAATTCCTACATTAGGGATTGTTGATTGA	this study	N/A

Supplementary Table 3. Plasmids and yeast strains.

Plasmid name	Baculovirus insect cell expression constructs
pZAJ-47	pIDC-*FLAG-STU2*
pZAJ-48	pACEBac-*His-SPC97*
pZAJ-49	pIDC-*His-SPC98*
pZAJ-66	pACEBac-*His-SPC97*/His-*SPC98*
pZAJ-50	pIDK-*TUB4*
pZAJ-51	pIDS-*His-SPC72*$^{1\text{-}599}$
pZAJ-70	pIDK-*TUB4*/*His-SPC72*$^{1\text{-}599}$
pZAJ-73	pIDS-*His-SPC72*$^{1\text{-}599:PA}$
pZAJ-74	pIDS-*His-SPC72*$^{1\text{-}599:3R}$
pZAJ-75	pIDK-*TUB4*/*His-SPC72*$^{1\text{-}599:PA}$
pZAJ-76	pIDK-*TUB4*/*His-SPC72*$^{1\text{-}599:3R}$
pZAJ-82	pACEBac-*FLAG-SPC97*
pZAJ-62	pIDC-*SPC98*
pZAJ-83	pACEBac-*FLAG-SPC97*/*SPC98*
pZAJ-81	pIDC-*His-GFP-MZT1*
pZAJ-44	pFastBac1-*FLAG-STU2*
pZAJ-104	pFastBac1-*FLAG-STU2*$^{\Delta 666\text{-}768}$
pZAJ-105	pFastBac1-*FLAG-STU2*$^{\Delta 894\text{-}924}$
pZAJ-107	pFastBac1-*FLAG-SPC98*
**Plasmid name**	***E. coli* plasmid constructs**
pZAJ-35	pET28b-*STU2*
pZAJ-45	pET28b-*SPC72*
pZAJ-57	pET28b-*His-SPC72*$^{1\text{-}599}$
pZAJ-87	pET28b-*His-SPC72*$^{1\text{-}239}$
pZAJ-88	pET28b-*His-SPC72*$^{1\text{-}290}$

pZAJ-89	pET28b-*His-SPC72*$^{291-599}$
pZAJ-97	pET28b-*His-GFP-SPC72*$^{230-290}$
pZAJ-98	pET28b-*His-GFP-SPC72*$^{300-350}$
pZAJ-99	pET28b-*His-GFP-SPC72*$^{291-428}$
pZAJ-100	pET28b-*His-GFP-SPC72*$^{430-480}$
pZAJ-108	pET28b-*His-GFP-SPC72*$^{429-599}$
pZAJ-109	pET28b-*His-GFP-SPC72*$^{300-350ELLY}$
pZAJ-110	pET28b-*His-GFP-SPC72*$^{430-480EDID1}$
pWM276	pETM41-*His-MBP-TEV-SPC72*$^{231-268}$
pWM277	pETM41-*His-MBP-TEV-SPC72*$^{231-268,3R}$
pWM280	pETM41-*GST-TEV-SPC72*$^{231-268}$
pWM281	pETM41-*GST-TEV-SPC72*$^{231-268,3R}$
pZAJ-111	pETM41-*His-MBP-FLAG-STU2*$^{882-924}$
pZAJ-112	pETM41-*His-MBP-FLAG-STU2*$^{882-924LIM}$
**Plasmid name**	***S. cerevisiae* plasmid constructs**
pZAJ-115	pRS315-*ScSPC72*
pZAJ-116	pRS315-*Scspc72*$^{\Delta P55-N62}$
pZAJ-117	pRS315-*Scspc72*^{3R}
pZAJ-118	pRS305-*ScSPC72*
pZAJ-119	pRS305-*Scspc72*$^{\Delta P55-N62}$
pZAJ-120	pRS305-*Scspc72*^{3R}
pMaM818 (Dr. M.Knop, ZMBH)	pFA6a-*IAA7-3xFLAG-Tubc6-kanMX*
pZAJ-121	pMM5-*CaSPC72*
pZAJ-122	pMM5-*Caspc72*$^{\Delta 300-350}$
pZAJ-123	pMM5-*Caspc72*$^{\Delta 430-480}$
pZAJ-124	pMM6-*CaSTU2*
pZAJ-125	pRS316-*GAL1-ScSPC72*

pZAJ-126	pRS316-*GAL1-Scspc72*^{3R}
pSM1026-1	pRS304-*GFP-TUB1*
**Yeast strains**	**Description**
ESM448-1, S288c background	*MATa ura3-52 lys2-801 ade2-101 trp1Δ63 his3Δ200 leu2Δ1 Δspc72::KanMx6* pRS316-*SPC72* [11]
YJP287-1, W303 background	*MATa ade2-1 ura3-1 his3-11, 15 trp1-1 leu2-3, 113 can1-100 ∆spc72::KanMx6* pRS316-*SPC72 SPC42-mCherry-hgh GFP-TUB1-ADE2*
ESM356-1	*MATa ura3-52 leu2Δ1 his3Δ200 trp1Δ63* [12]
DR337-1, ESM356-1 background	*MATa ura3-52::pRS306-pADH1-OsTIR1-9Myc lys2-801 ade2-101 trp1Δ63 his3Δ200 leu2Δ1 SPC42-mCherry-hphNT1*
YAJZ023 (this study), modified from DR337-1	*MATa ura3-52::pRS306-pADH1-OsTIR1-9Myc lys2-801 ade2-101 trp1Δ63::pRS304-GFP-TUB1 his3Δ200 leu2Δ1 SPC42-mCherry-hphNT1 SPC72-IAA7-3xFLAG-Tubc6-kanMX* (*Tubc6 stands for Terminator of the *UBC6* gene)

Supplementary Table 4. Experimental details of HX-MS Experiments.

Data Set	His-Spc72$^{291-599}$ + FLAG-Stu2 (1:1, 1:2)	FLAG-Stu2 + His-Spc72$^{291-599}$ (1:1, 1:3)
HDX reaction details	25 mM Tris-HCl, pH 8, 150 mM $NaCl_2$, 0.5 mM $MgCl_2$, 0.5 mM EGTA (buffer was prepared as H_2O buffer and lyophilised and resuspended in D_2O three times)	
HDX time course (s)	30	30
HDX control samples	Maximally labeled control His-Spc72$^{291-599}$)	Maximally labeled control (FLAG-Stu2)
Back-exchange (mean / IQR)	31.1% / 7.5%	30.2%/7%
# of Peptides	24	42
Sequence coverage	80.3%	56.9%
Average peptide length / Redundancy	16.2/1.3	17/1.4
Replicates (biological or technical)	2 (technical)	2 (technical)
Repeatability	0.22 (average standard deviation)	0.6 (average standard deviation)
Significant differences in HDX (delta HDX > X D)	1.05 D (95% CI)	2.9 D (95%CI)

Supplementary References

1 Gunzelmann, J. *et al.* The microtubule polymerase Stu2 promotes oligomerization of the gamma-TuSC for cytoplasmic microtubule nucleation. *Elife* **7** (2018). https://doi.org:10.7554/eLife.39932

2 Dendooven, T. *et al.* Structure of the native gamma-tubulin ring complex capping spindle microtubules. *Nat Struct Mol Biol* (2024). https://doi.org:10.1038/s41594-024-01281-y

3 Zupa, E. *et al.* The cryo-EM structure of a gamma-TuSC elucidates architecture and regulation of minimal microtubule nucleation systems. *Nat Commun* **11**, 5705 (2020). https://doi.org:10.1038/s41467-020-19456-8

4 Brilot, A. F. *et al.* CM1-driven assembly and activation of yeast gamma-tubulin small complex underlies microtubule nucleation. *Elife* **10** (2021). https://doi.org:10.7554/eLife.65168

5 Goddard, T. D. *et al.* UCSF ChimeraX: Meeting modern challenges in visualization and analysis. *Protein Sci* **27**, 14-25 (2018). https://doi.org:10.1002/pro.3235

6 Lin, T. C. *et al.* MOZART1 and gamma-tubulin complex receptors are both required to turn gamma-TuSC into an active microtubule nucleation template. *J Cell Biol* **215**, 823-840 (2016). https://doi.org:10.1083/jcb.201606092

7 Wieczorek, M. *et al.* Asymmetric Molecular Architecture of the Human gamma-Tubulin Ring Complex. *Cell* **180**, 165-175 e116 (2020). https://doi.org:10.1016/j.cell.2019.12.007

8 Wieczorek, M., Huang, T. L., Urnavicius, L., Hsia, K. C. & Kapoor, T. M. MZT Proteins Form Multi-Faceted Structural Modules in the gamma-Tubulin Ring Complex. *Cell Rep* **31**, 107791 (2020). https://doi.org:10.1016/j.celrep.2020.107791

9 Elfmann, C. & Stülke, J. PAE viewer: a webserver for the interactive visualization of the predicted aligned error for multimer structure predictions and crosslinks. *Nucleic Acids Research* **51**, W404-W410 (2023). https://doi.org:10.1093/nar/gkad350

10 Wurtz, M. *et al.* Reconstitution of the recombinant human gamma-tubulin ring complex. *Open Biol* **11**, 200325 (2021). https://doi.org:10.1098/rsob.200325

11 Knop, M. & Schiebel, E. Receptors determine the cellular localization of a γ-tubulin complex and thereby the site of microtubule formation. *The EMBO Journal* **17**, 3952-3967 (1998).

12 Pereira, G., Tanaka, T. U., Nasmyth, K. & Schiebel, E. Modes of spindle pole body inheritance and segregation of the Bfa1p-Bub2p checkpoint protein complex. *Embo j* **20**, 6359-6370 (2001). https://doi.org:10.1093/emboj/20.22.6359
