# Supplementary material for: Structural insights into the interplay between microtubule polymerases, γ-tubulin complexes and their receptors
Source: Nat Commun. 2025 Jan 5;16:402. doi: 10.1038/s41467-024-55778-7 (PMC11701102; doi:10.1038/s41467-024-55778-7)
Supplement: Supplementary file 2 — Reporting Summary [file 41467_2024_55778_MOESM2_ESM.pdf]

nature portfolio

Corresponding author(s): Stefan Pfeffer, Elmar Schiebel

Last updated by author(s): Dec 9, 2024

# Reporting Summary

Nature Portfolio wishes to improve the reproducibility of the work that we publish. This form provides structure for consistency and transparency in reporting. For further information on Nature Portfolio policies, see our Editorial Policies and the Editorial Policy Checklist.

## Statistics

For all statistical analyses, confirm that the following items are present in the figure legend, table legend, main text, or Methods section.

| n/a | Confirmed | |
|---|---|---|
| ☐ | ☒ | The exact sample size (*n*) for each experimental group/condition, given as a discrete number and unit of measurement |
| ☐ | ☒ | A statement on whether measurements were taken from distinct samples or whether the same sample was measured repeatedly |
| ☐ | ☒ | The statistical test(s) used AND whether they are one- or two-sided<br>*Only common tests should be described solely by name; describe more complex techniques in the Methods section.* |
| ☒ | ☐ | A description of all covariates tested |
| ☒ | ☐ | A description of any assumptions or corrections, such as tests of normality and adjustment for multiple comparisons |
| ☐ | ☒ | A full description of the statistical parameters including central tendency (e.g. means) or other basic estimates (e.g. regression coefficient) AND variation (e.g. standard deviation) or associated estimates of uncertainty (e.g. confidence intervals) |
| ☐ | ☒ | For null hypothesis testing, the test statistic (e.g. *F*, *t*, *r*) with confidence intervals, effect sizes, degrees of freedom and *P* value noted<br>*Give P values as exact values whenever suitable.* |
| ☒ | ☐ | For Bayesian analysis, information on the choice of priors and Markov chain Monte Carlo settings |
| ☒ | ☐ | For hierarchical and complex designs, identification of the appropriate level for tests and full reporting of outcomes |
| ☒ | ☐ | Estimates of effect sizes (e.g. Cohen's *d*, Pearson's *r*), indicating how they were calculated |

*Our web collection on statistics for biologists contains articles on many of the points above.*

## Software and code

Policy information about availability of computer code

| | |
|---|---|
| Data collection | Cryo-EM data was collected using EPU (version 2.6) on a Krios TEM equipped with a Gatan K3 camera operated by Gatan Microscopy Suite (version 3.32). Negative stain EM data was collected on a Talos L120C equipped with a Ceta 16M using Thermo Fisher Scientific EPU version 2.9. Size exclusion chromatography (Superose 6 increase 10/300 GL, GE healthcare) was performed on an ÄKTA go operated using Unicorn (version 7.6) for acquiring the SEC data. The Monolith NT.115 instrument (NanoTemper Technologies) was used for the MST measurements and data collection. SDS PAGE images were acquired with LAS4000IR v2.1. The DeltaVision RT system (Applied Precision) on an Olympus IX71 microscope equipped with 100X NA UPlanSAPO objective lens (Olympus) was used for image acquisition in yeast experiments. A CanoScan 5600F scanning machine running MP Navigator EX - CanoScan 5600F software (Canon U.S.A., Inc.) was used for yeast dish imaging and SDS PAGE imaging. Mass photometry was measured using Refeyn TwoMP mass photometer (Refeyn Ltd, Oxford, UK). SEC-MALS was performed on an Agilent 1260 Infinity II HPLC system (Agilent) coupled to a MALS system (MiniDAWN and Optilab, Wyatt Technology) installed with a Superdex 200 increase 5/150 GL gel-filtration column (Cytiva) to collect the Mass photometer related data. Mass spectrometry data were collected on a MaXis QTOF mass spectrometer (Bruker Daltonics, Bremen, Germany). |
| Data analysis | EM data were processed using Relion 3.0 and 3.1, cryoSPARC 3.2, MotionCor2 v1.0.5 and Gctf 1.06. All atomic model and density-related analysis and visualtisation was done using UCSF Chimera 1.14 and UCSF ChimeraX 1.3. Model building was performed using ModelAngelo 0.2.4, Coot 0.9.2, Phenix 1.20 and ISOLDE 1.3. AlphaFold predictions were carried out using AlphaFold Multimer 2.3. Curve visualisation was done using R 4.1.2 in RStudio 2022.02.0 and Prism 9.1. For SEC data visualisation, GraphPad PRISM software (GraphPad v.10) was used. For MST data, normalised fluorescence readings (thermophoresis plus T-jump) were plotted to generate binding curves by using the NanoTemper software and then the curves were exported into R for visualisation. The images obtained from DeltaVision RT system concerning the yeast experiments, the processing and analysis were performed with the open-source software package ImageJ 1.46r (National Institutes of Health). GraphPad PRISM software (GraphPad v.10.0) was used for statistical analysis of the yeast phenotypes with a 2-way ANOVA test (Šídák's multiple comparisons test). Mass photometry data were analysed using Refeyn DiscoverMP 2024 R1 software (Refeyn Ltd, Oxford, UK). SEC- |

MALS data were analysed using Astra 8.2.2 software (Wyatt Technology). Mass spectrometry data were analyzed using the Analysis software of the MaXis mass spectrometer. Statistical analysis of mass spectrometry data was performed using Prism 9 version 9.5.1 All HDX-MS data are corrected for back exchange. Protein band intensities on Western blots or Coomassie blue-stained gels were quantified using ImageJ.

For manuscripts utilizing custom algorithms or software that are central to the research but not yet described in published literature, software must be made available to editors and reviewers. We strongly encourage code deposition in a community repository (e.g. GitHub). See the Nature Portfolio guidelines for submitting code & software for further information.

## Data

Policy information about availability of data

All manuscripts must include a data availability statement. This statement should provide the following information, where applicable:

- Accession codes, unique identifiers, or web links for publicly available datasets
- A description of any restrictions on data availability
- For clinical datasets or third party data, please ensure that the statement adheres to our policy

Atomic coordinates and the associated cryo-EM densities generated in this study have been deposited in the Protein Data Bank and the Electron Microscopy Data Bank under accession codes PDB-9H9Q [https://www.rcsb.org/structure/9H9Q] / EMD-51971 [https://www.ebi.ac.uk/emdb/EMD-51971] (γ-TuSC in ring-like oligomer), PDB-9H9R [https://www.rcsb.org/structure/9H9R] / EMD-51972 [https://www.ebi.ac.uk/emdb/EMD-51972] (14-spoked γ-TuRC) and PDB-9H9P [https://www.rcsb.org/structure/9H9P] (human CDK5RAP2CM1 in complex with GCP2/GCP6/Mzt2). The mass spectrometry proteomics data generated in this study have been deposited to the ProteomeXchange Consortium via the PRIDE partner repository with the dataset identifier PXD057184 [https://proteomecentral.proteomexchange.org/cgi/GetDataset?ID=PXD057184]. Models predicted by AlphaFold-Multimer generated in this study have been deposited to the ModelArchive (https://modelarchive.org/) under accession codes ma-eefkg [https://modelarchive.org/doi/10.5452/ma-eefkg], ma-bktlp [https://modelarchive.org/doi/10.5452/ma-bktlp], ma-kzdh2 [https://modelarchive.org/doi/10.5452/ma-kzdh2] and ma-3kpdx [https://modelarchive.org/doi/10.5452/ma-3kpdx]. Cryo-EM data collection, refinement, and validation statistics data, experimental details for HX-MS experiments as well as primer, plasmid, and yeast strains are provided in the Supplementary Information file. Source data are provided in this paper. Constructs generated in this study are available upon request to the corresponding authors. Published structural data used in this article: PDB-5W3F [https://www.rcsb.org/structure/5W3F], PDB-6V6S [https://www.rcsb.org/structure/6V6S], PDB-6X0V [https://www.rcsb.org/structure/6X0V], PDB-7ANZ [https://www.rcsb.org/structure/7ANZ], PDB-7M2Y [https://www.rcsb.org/structure/7M2Y], PDB-7M2Z [https://www.rcsb.org/structure/7M2Z], PDB-8QV2 [https://www.rcsb.org/structure/8QV2], PDB-8QV3 [https://www.rcsb.org/structure/8QV3], EMD-8758 [https://www.ebi.ac.uk/emdb/EMD-8758], EMD-21985 [https://www.ebi.ac.uk/emdb/EMD-21985].

## Research involving human participants, their data, or biological material

Policy information about studies with human participants or human data. See also policy information about sex, gender (identity/presentation), and sexual orientation and race, ethnicity and racism.

| | |
|---|---|
| Reporting on sex and gender | No experiments with human participants were performed. |
| Reporting on race, ethnicity, or other socially relevant groupings | No experiments with human participants were performed. |
| Population characteristics | No experiments with human participants were performed. |
| Recruitment | No experiments with human participants were performed. |
| Ethics oversight | No experiments with human participants were performed. |

Note that full information on the approval of the study protocol must also be provided in the manuscript.

# Field-specific reporting

Please select the one below that is the best fit for your research. If you are not sure, read the appropriate sections before making your selection.

☒ Life sciences ☐ Behavioural & social sciences ☐ Ecological, evolutionary & environmental sciences

For a reference copy of the document with all sections, see nature.com/documents/nr-reporting-summary-flat.pdf

# Life sciences study design

All studies must disclose on these points even when the disclosure is negative.

| | |
|---|---|
| Sample size | For cryo-EM data, no statistical method was chosen to determine the sample size. The number of micrographs was chosen to obtain a number of particles sufficient to reconstruct a detailed 3D density of the gamma-TuSC in complex with Spc72. Collected cryo-EM data contained 33781 images of gamma-TuSC in complex with Spc72, corresponding to two three-day microscopy sessions with parameters specified in the method section. The initial number of picked particles was 1711419. After multiple rounds of 3D classification, we reached a final dataset of 129908 (single γTuSC within higher oligomer) or 8261 (full 14-spoked ring) particles that were used for refinement in RELION. The final reconstructions reached a resolution that was estimated by the Gold Standard FSC method. AlphaFold predictions produced 5x5 models per construct. For negative stain EM data, no statistical method was chosen to determine the sample size. The number of micrographs was chosen to obtain a number of particles sufficient for 2D and 3D class averaging. In total, 609 images of γ-TuSC/His-Spc72(1-599)/FLAG-Stu2 sample in TBS buffer, 726 images of γ-TuSC/ His-Spc72(1-599)/FLAG-Stu2 and 2481 images of γ-TuSC/ His-Spc72(1-599)/His-GFP-Mzt1 were acquired. For in vivo and light microscopy experiments, no statistical method was used to predict the sample size. For each biological replicate, at least 10 images were taken, each containing at least 5 cells to achieve a sufficient number of cells to analyze. Live cell imaging was |

| | |
|---|---|
| | conducted by imaging at least 50 cells per condition. The sample size was sufficient for negative stain EM, cryo-EM and light microscopy analyses. For MST, 16 samples were used for each measurement. |
| Data exclusions | No data exclusions were performed. All collected negative stain EM and cryo-EM images were used for particle picking. Cryo-EM particle selection was performed in several 3D classification rounds, and the quality of resulting 3D maps was the exclusion criterion, as is standard image processing practice in the cryo-EM. Negative stain EM particle selection was performed in several 2D classification rounds and the exclusion criterion was based on the shape of the class averages. For fluorescence microscopy experiments, all images containing cells were used for analysis. For MST, SEC, SEC-MALS, Mass photometry and HX-MS, no data were excluded and all the data were used for the analysis. |
| Replication | All the replication times mean the biological replicates if not specifically addressed in this section:<br>Negative stain EM data acquisition was performed at least once, and SDS page analysis were performed at least twice for protein purifications. For in vivo experiments, at least two independent experiments were conducted to confirm the reproducibility of the experiment. For live-cell imaging and DAPI staining, at least two independent experiments were conducted to confirm the reproducibility of the experiment. For FLAG-IP experiments, at least 2 independent experiments were performed. Protein expression and purification were performed in at least 3 replicates. SEC (Size-exclusion chromatography) experiments were performed at least twice. SEC-MALS experiment was performed once with 0 repetitions. MST measurement was performed twice. The protein band intensity quantification was performed independently 3 times. Mass photometry measurements were performed once or twice. All independent experiments were successfully replicated for the biological replication experiments.<br>Cryo-EM data were acquired in three sessions on two grids. For cryo-EM and negative stain EM analysis, the final 2D classes and 3D densities are averages of hundreds to thousands of particles and thus repetitions of the experiments were not necessary. Alphafold predictions produce five predicted models per five random seeds as per the default settings. All HX-MS experiments were conducted technical two replicates. Only peptic peptides are presented that could be detected in each replicate with sufficient intensity and for which we obtained data for the 100% control. |
| Randomization | Positions for image acquisition in cryo-EM and Negative stain EM were selected based on the presence of the particles. Positions for image acquisition in live microscopy experiments were selected on the presence of yeast cells/microtubules/SPB/nuclei. A wide field of view was used to image a sufficienct number of cells/microtubules/SPB/nuclei per image to avoid biased image seclection. Other experiments were not related to randomization. |
| Blinding | Cryo-EM, negative stain EM analysis and AlphaFold predictions were not blinded because it is performed computationally. For other experiments like cloning and expression protein purification, it was technically not possible; In other cases, the findings were confirmed by independent approaches e.g. IP experiments. For live-cell fluorescence experiments, it was not feasible to apply blinding experiments because of large sample size. Other experiments of SEC, Mass photometry, HX-MS and SEC-MALS were not related to blinding. |

# Reporting for specific materials, systems and methods

We require information from authors about some types of materials, experimental systems and methods used in many studies. Here, indicate whether each material, system or method listed is relevant to your study. If you are not sure if a list item applies to your research, read the appropriate section before selecting a response.

## Materials & experimental systems

| n/a | Involved in the study |
|---|---|
| ☐ | ☒ Antibodies |
| ☐ | ☒ Eukaryotic cell lines |
| ☒ | ☐ Palaeontology and archaeology |
| ☒ | ☐ Animals and other organisms |
| ☒ | ☐ Clinical data |
| ☒ | ☐ Dual use research of concern |
| ☒ | ☐ Plants |

## Methods

| n/a | Involved in the study |
|---|---|
| ☒ | ☐ ChIP-seq |
| ☒ | ☐ Flow cytometry |
| ☒ | ☐ MRI-based neuroimaging |

## Antibodies

| | |
|---|---|
| Antibodies used | Primary antibodies used in this study for Immunoblots were:<br>Anti-FLAG antibody (mouse; clone 9A3, 1:1000; Cell Signaling); Anti-penta-His antibody (mouse, 1:2000; Qiagen 34660); Anti-N-S. cerevisiae Spc72 (rabbit, 1:300; homemade); Anti-GAPDH antibody (mouse, 1:10000; Proteintech®); Anti-γ-tubulin antibody (guinea pig; 1:1000; home-made; C. albicans γ-tubulin antigen (331-498aa fragment) was sent to Eurogentec for antibody production).<br>Secondary antibodies used in this study for Immunoblots were:<br>Anti-Mouse HRP-conjugated IgG (H+L) (donkey, 1:5000, JacksonImmunoResearch EUROPE LTD.); Anti-Guinea pig HRP-conjugated IgG (H+L) (donkey, 1:5000, JacksonImmunoResearch EUROPE LTD.); Anti-Rabbit HRP-conjugated IgG (H+L) (donkey, 1:5000, JacksonImmunoResearch EUROPE LTD.) |
| Validation | Primary antibodies used in this study for Immunoblots were:<br>Anti-FLAG antibody (https://www.cellsignal.com/products/primary-antibodies/dykddddk-tag-9a3-mouse-mab-binds-to-same-epitope-as-sigma-s-anti-flag-m2-antibody/8146); Anti-penta-His antibody (https://www.qiagen.com/us/products/discovery-and-translational-research/protein-purification/tagged-protein-expression-purification-detection/anti-his-antibodies-bsa-free); Anti-N-Spc72 (S. cerevisiae): see reference Gunzelmann et al., 2018, The microtubule polymerase Stu2 promotes oligomerization of the γ-TuSC for cytoplasmic microtubule nucleation. Elife. 2018 Sep 17;7:e39932. doi: 10.7554/eLife.39932;<br>Anti-GAPDH antibody (https://www.ptglab.com/products/GAPDH-Antibody-60004-1-Ig.htm). |

Secondary antibodies used in this study for Immunoblots were:
Anti-Mouse HRP-conjugated IgG (H+L) (https://www.jacksonimmuno.com/catalog/products/715-035-151);
Anti-Guinea pig HRP-conjugated IgG (H+L) (https://www.jacksonimmuno.com/catalog/products/706-035-148);
Anti-Rabbit HRP-conjugated IgG (H+L) (https://www.jacksonimmuno.com/catalog/products/711-035-152).

## Eukaryotic cell lines

Policy information about [cell lines and Sex and Gender in Research](#)

Cell line source(s)

SF21 insect cell line was obtained from the EMBL protein expression facility. Sf9 insect cells (Spodoptera frugiperda) Sigma-Aldrich. S. cerevisiae S288c ESM448-1 strain (MATa ura3-52 lys2-801 ade2-101 trp1Δ63 his3Δ200 leu2Δ1 Δspc72::KanMx6pRS316-SPC72). The strain was modified based on the FY1679 strain, which is a diploid made from haploid parents FY23 (mating type a) and FY73 (mating type α) by Fred Winston and colleagues who used gene replacement to develop a set of yeast strains isogenic to S288C but repaired for GAL2, and which also contained nonreverting mutations in several genes commonly used for selection in the laboratory environment (URA3, TRP1, LYS2, LEU2, HIS3). See reference of Foury F, et al. (1998) The complete sequence of the mitochondrial genome of Saccharomyces cerevisiae. FEBS Lett 440 (3):325-31 PMID:9872396. S. cerevisiae YJP287-1 strain (MATa ade2-1 ura3-1 his3-11, 15 trp1-1 leu2-3, 113 can1-100 Δspc72::KanMx6 pRS316-SPC72 SPC42-mCherry-hgh GFP-TUB1-ADE2) in the W303 background. The W303 strain was from Susan Wente. ESM356-1 strain (MATa ura3-52 leu2Δ1 his3Δ200 trp1Δ63), the strain was from the previous work, see reference of Pereira G, et al., Modes of spindle pole body inheritance and segregation of the Bfa1p-Bub2p checkpoint protein complex. EMBO J 2001, 20 (22), 6359-6370. DOI:10.1093/emboj/20.22.6359. DR337-1 strain (MATa ura3-52::pRS306-pADH1-OsTIR1-9Myc lys2-801 ade2-101 trp1Δ63 his3Δ200 leu2Δ1 SPC42-mCherry-hphNT1), ESM356 background, which was made in Dr. E.Schiebel's group, ZMBH. YAJZ023 ( MATa ura3-52::pRS306-pADH1-OsTIR1-9Myc lys2-801 ade2-101 trp1Δ63::pRS304-GFP-TUB1 his3Δ200 leu2Δ1 SPC42-mCherry-hphNT1 SPC72-IAA7-3xFLAG-Tubc6-kanMX (* Tubc6 stands for "Terminator of the Ubc 6 gene")), this study, modified from the strain of DR337-1.

Authentication

Cell lines were examined for their morphology by microscopy.

Mycoplasma contamination

Mycoplasma test was negative for all cell lines.

Commonly misidentified lines
(See [ICLAC](#) register)

No misidentified cell lines in this study.

## Plants

Seed stocks

No experiments with plants were performed.

Novel plant genotypes

No experiments with plants were performed.

Authentication

No experiments with plants were performed.
