# Supplementary material for: Structural insights into the interplay between microtubule polymerases, γ-tubulin complexes and their receptors
Source: Nat Commun. 2025 Jan 5;16:402. doi: 10.1038/s41467-024-55778-7 (PMC11701102; doi:10.1038/s41467-024-55778-7)

Source data, SDS-Page gels

Related to Fig. 1a

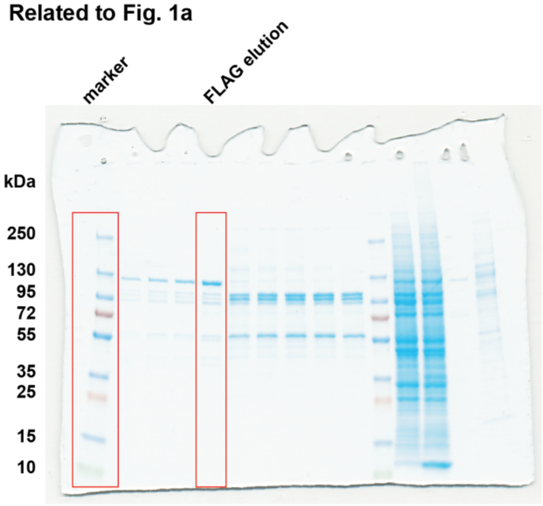

Related to Supplementary Fig. 2a

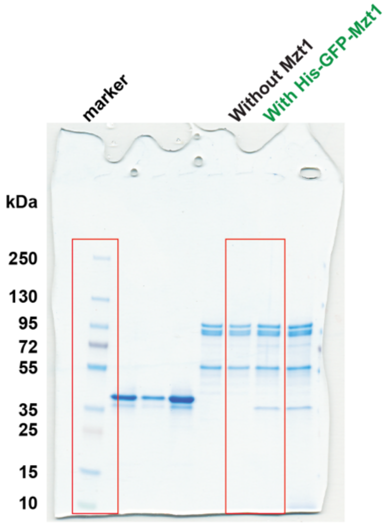

Related to Supplementary Fig. 1d

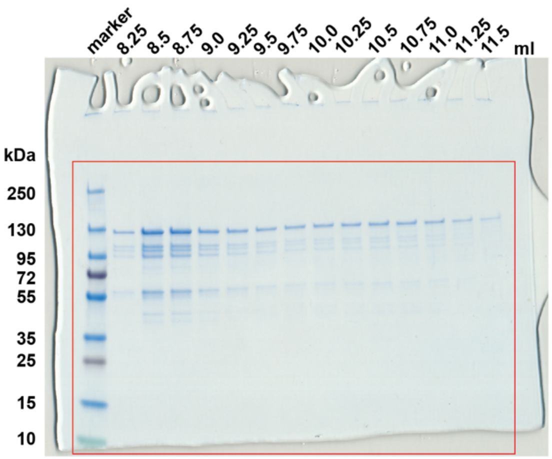

Related to Supplementary Fig. 2c (Upper)

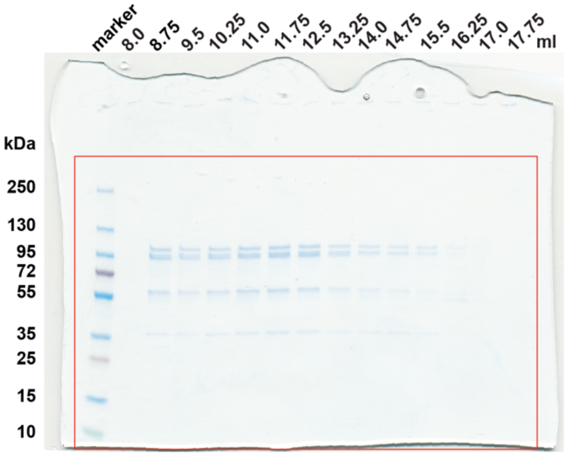

Related to Supplementary Fig. 2c (Bottom)

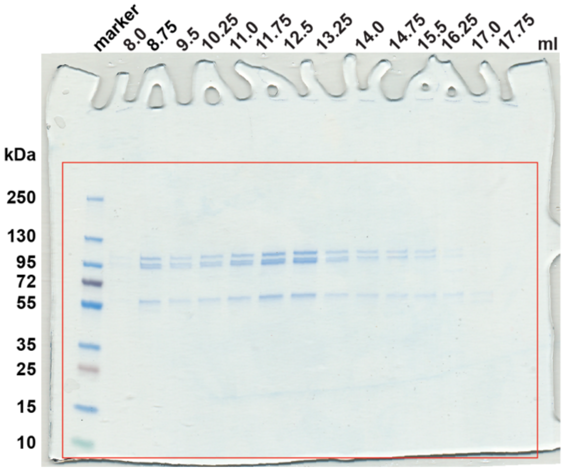

Source data, SDS-Page gels

Related to Supplementary Fig.1f

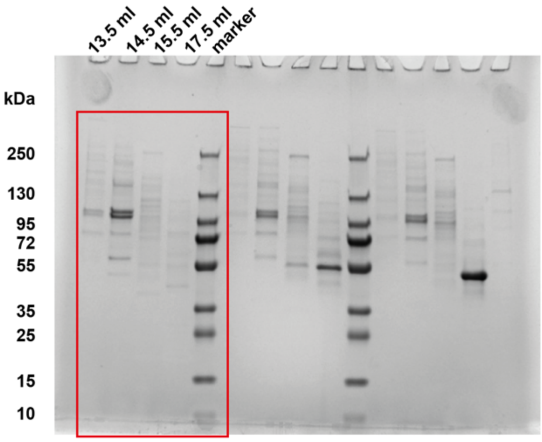

Related to Supplementary Fig.9a

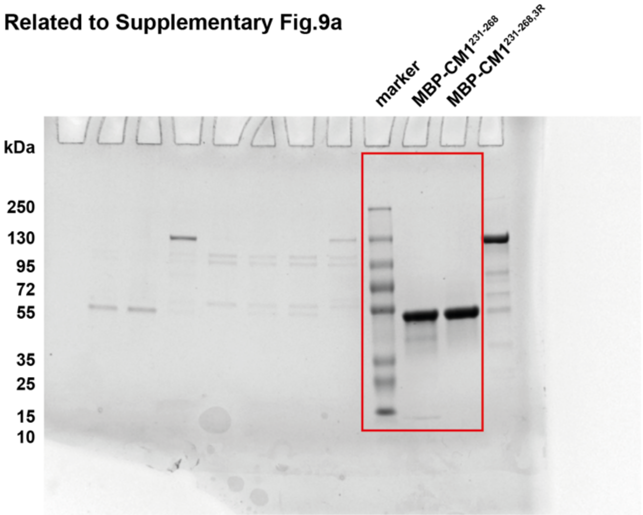

Related to Supplementary Fig.9e

Related to Supplementary Fig.15a

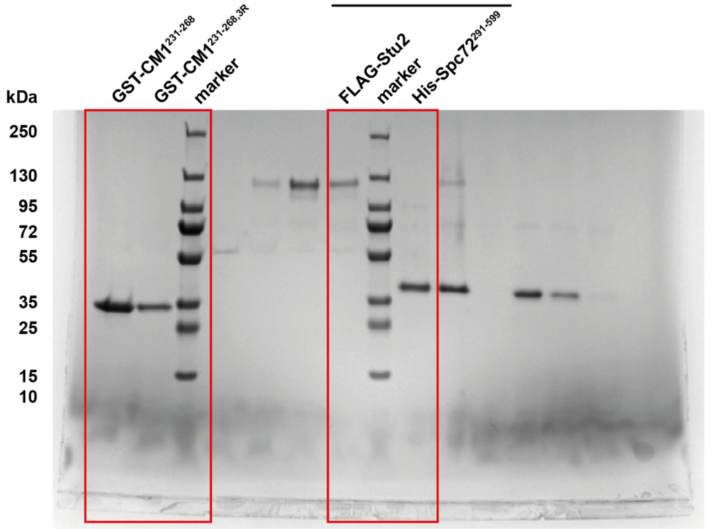

Source data, immunoblots

Related to Fig. 3e (γ-TuSC/His-Spc72$^{1-599}$/FLAG-Stu2, upper)

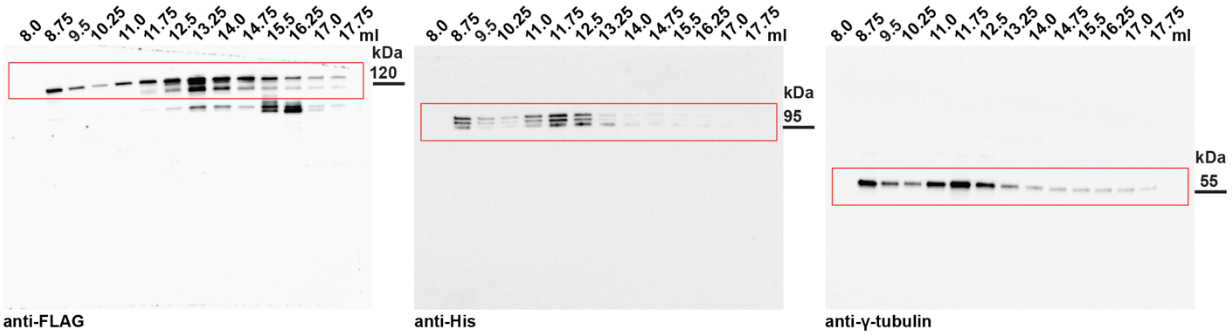

Related to Fig. 3e (γ-TuSC/His-Spc72$^{1-599,PA}$/FLAG-Stu2, bottom)

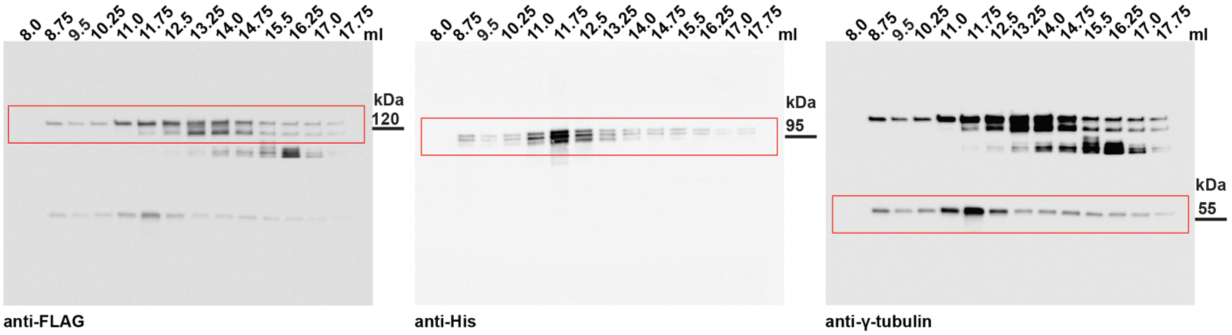

Related to Fig. 4f

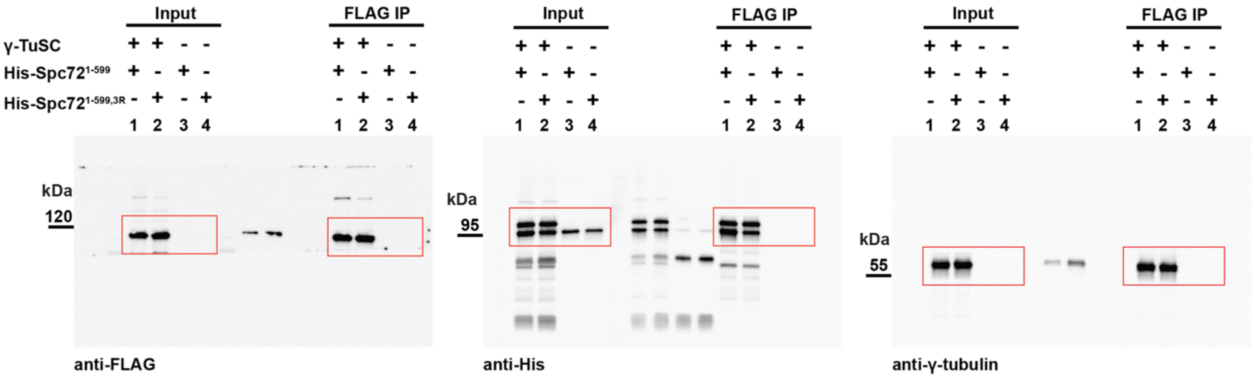

Source data, immunoblots

Related to Fig. 4h (γ-TuSC/His-Spc72$^{1\text{-}599,3R}$/FLAG-Stu2)

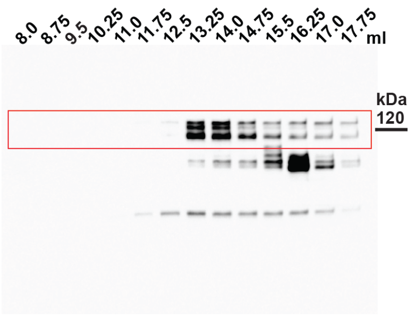

anti-FLAG

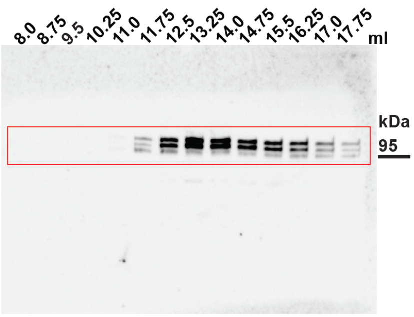

anti-His

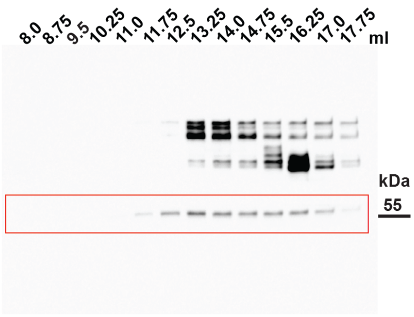

anti-γ-tubulin

Related to Fig. 4h (His-Spc72$^{1\text{-}599,3R}$ after SEC)

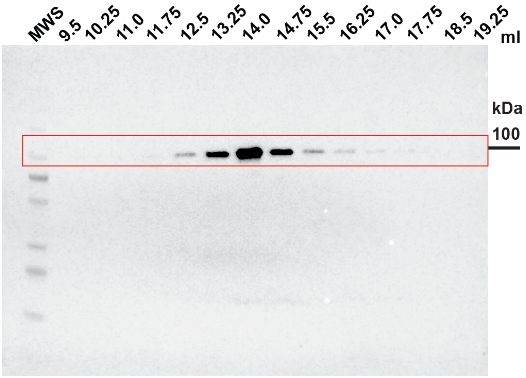

anti-His

Related to Fig. 5d

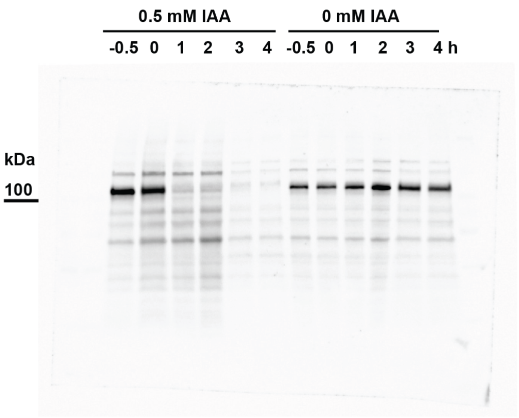

anti-N-Spc72 (*S. cerevisiae*)

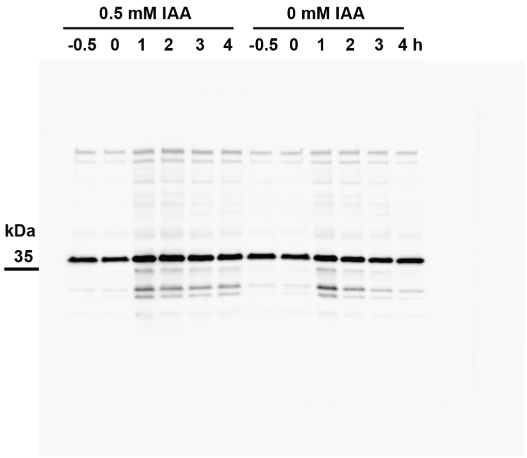

anti-GAPDH

Source data, immunoblots

Related to Fig. 6a

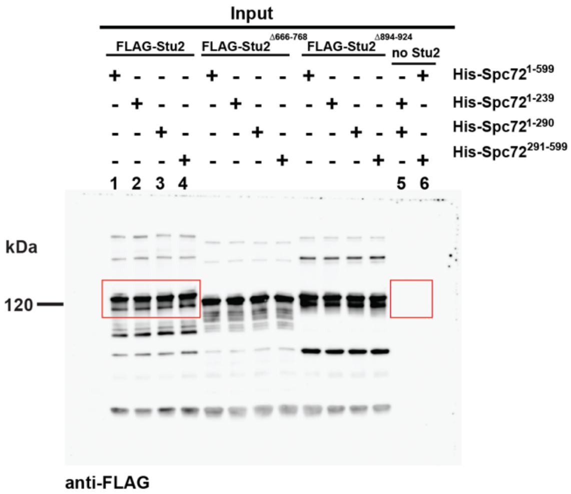

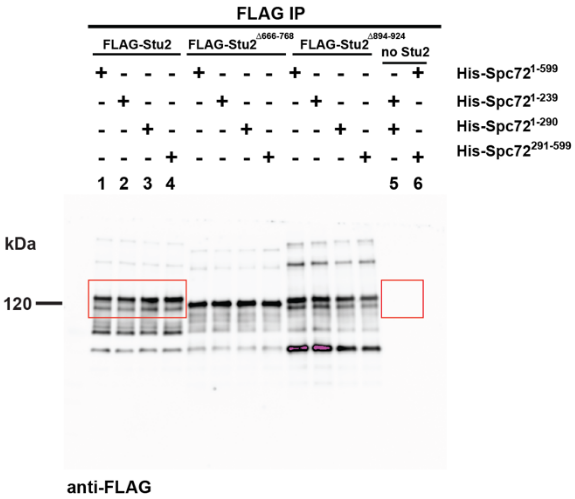

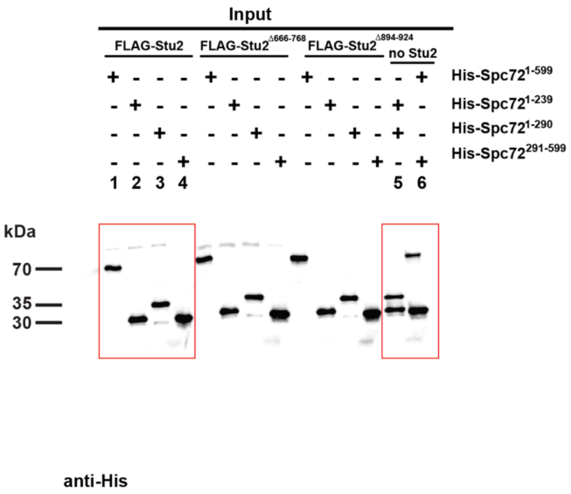

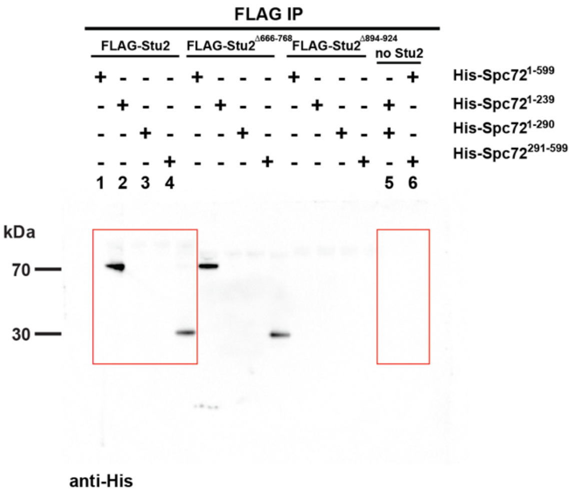

Source data, immunoblots

Related to Fig. 6b

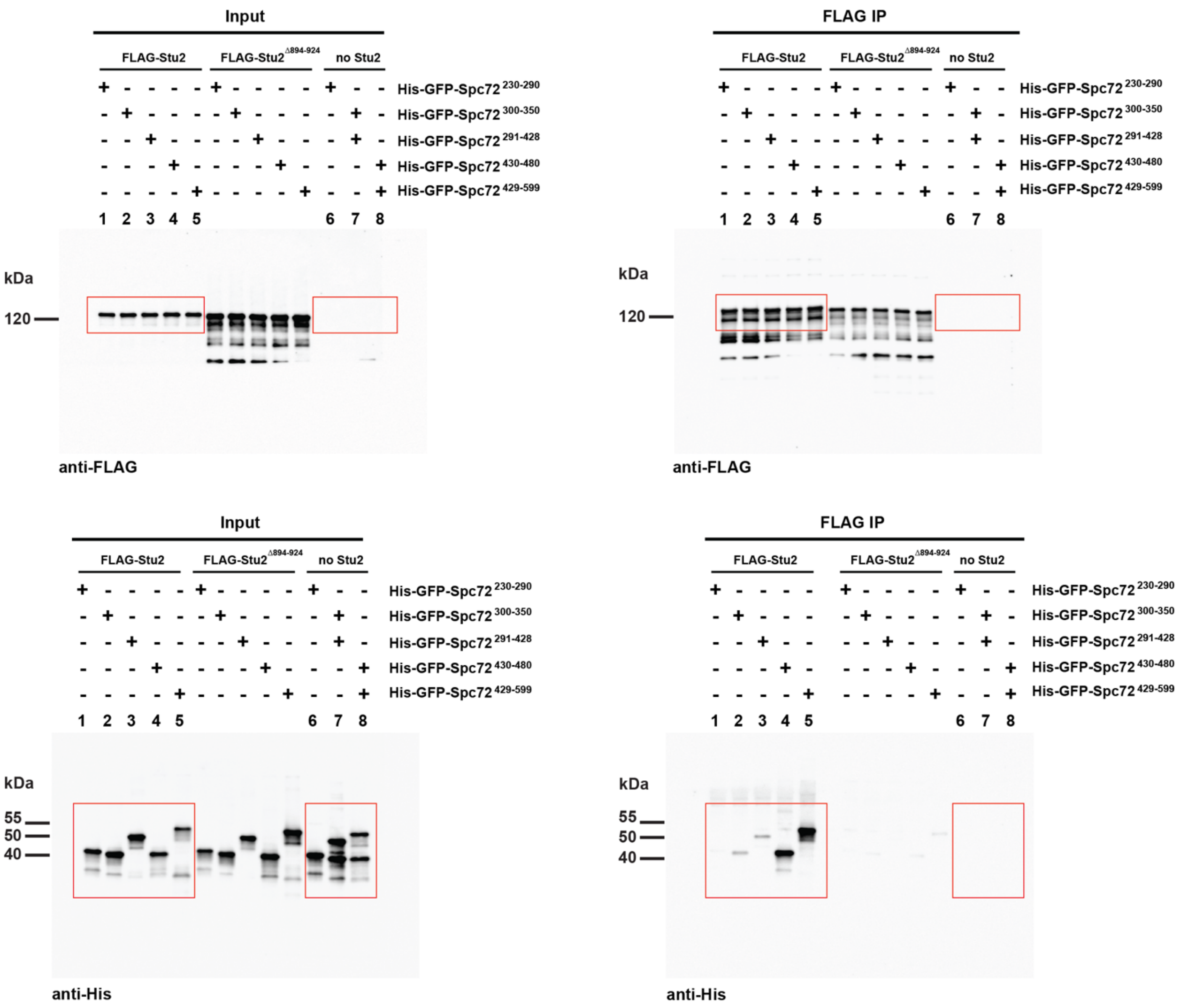

Source data, immunoblots

Related to Supplementary Fig. 1b

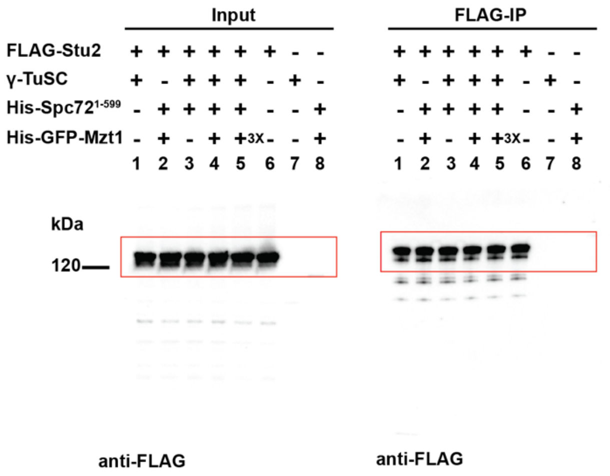

Related to Supplementary Fig. 1b

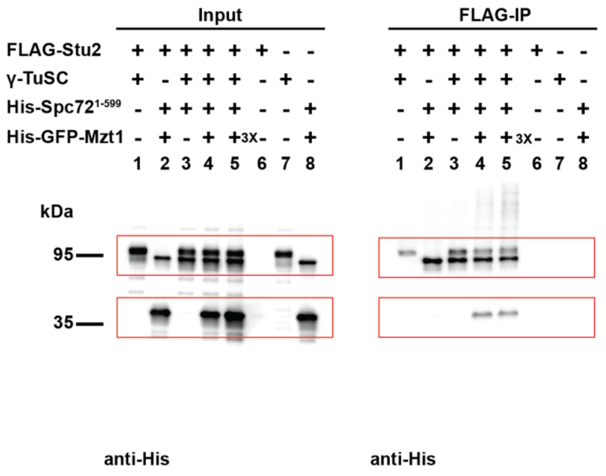

Related to Supplementary Fig. 1b

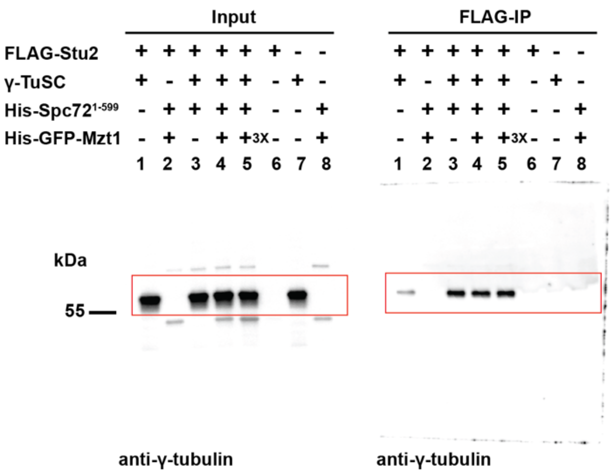

Related to Supplementary Fig. 2c

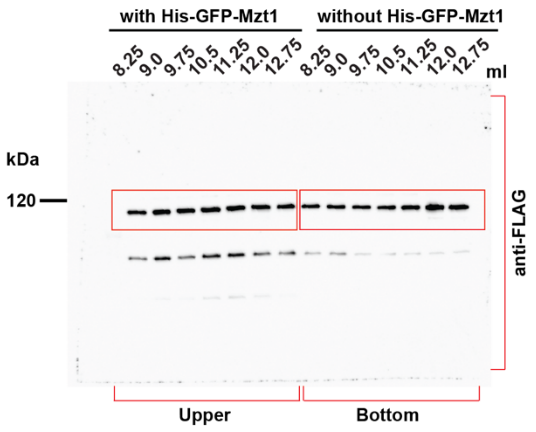

Related to Supplementary Fig. 2c

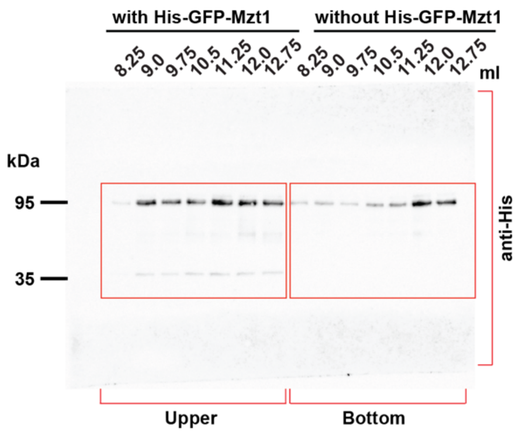

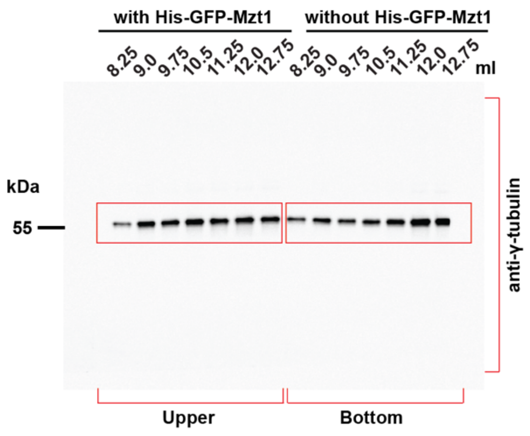

Source data, immunoblots

Related to Supplementary Fig. 10b

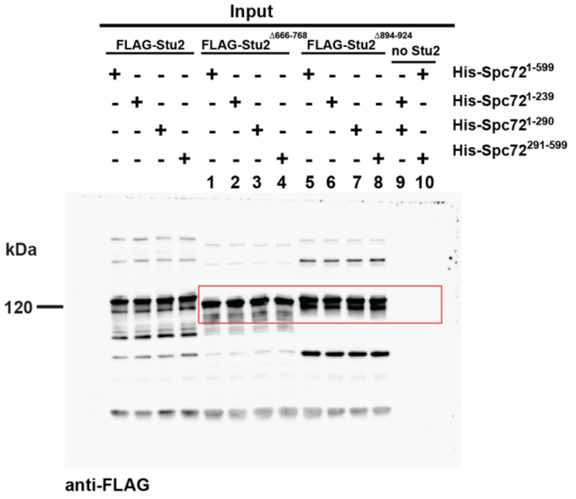

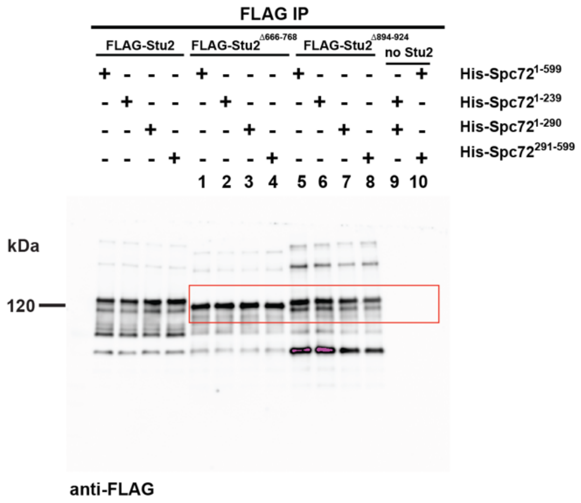

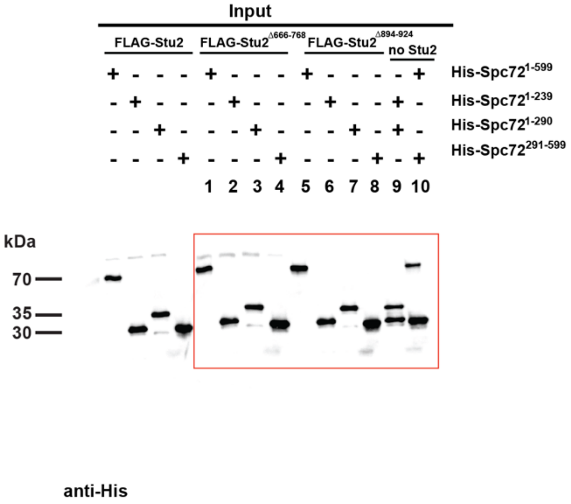

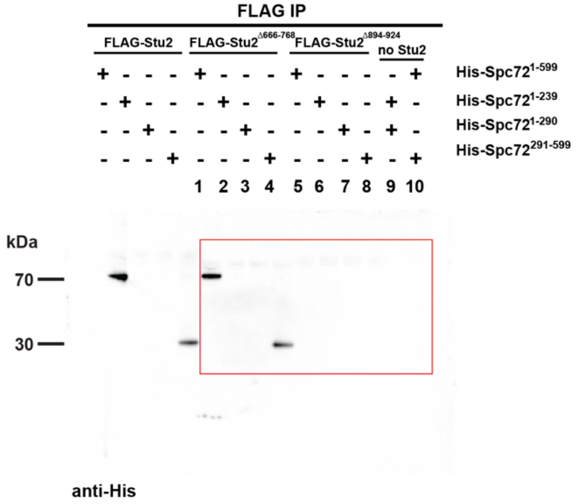

Source data, immunoblots

Related to Supplementary Fig. 10c

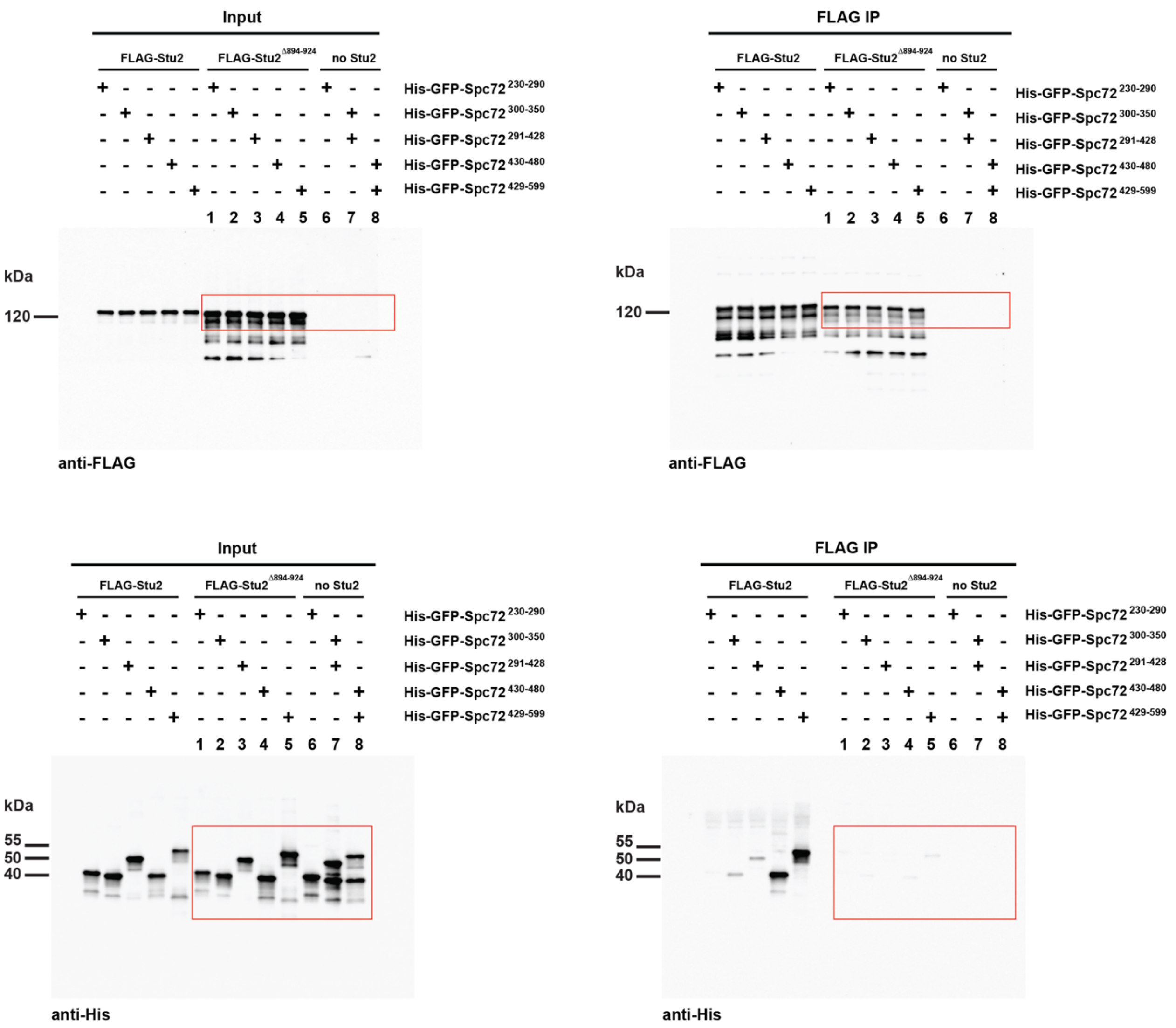

Source data, immunoblots

Related to Supplementary Fig. 10d

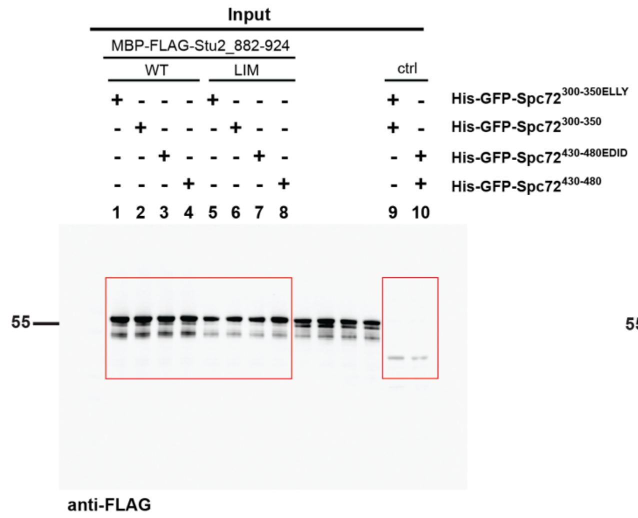

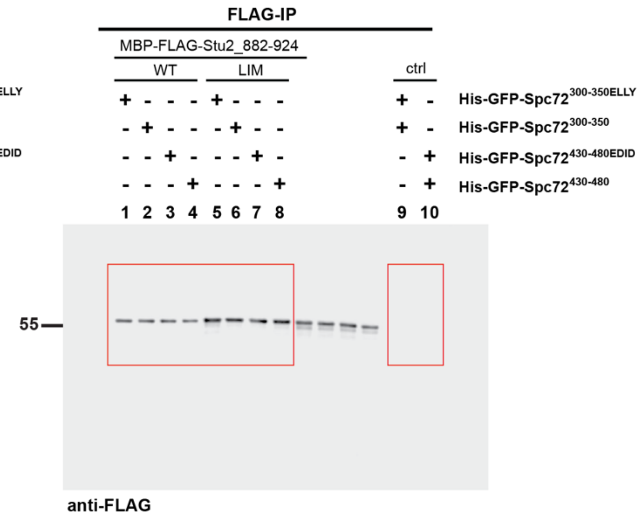

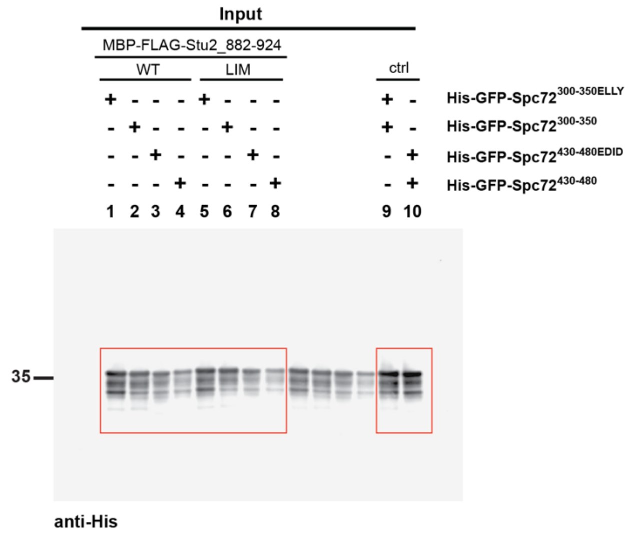

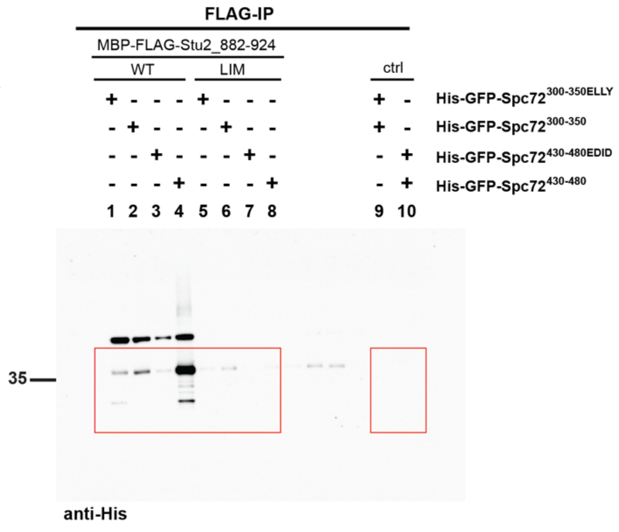

Source data, immunoblots

Related to Fig. 4f γ-TuSC$^{FLAG\text{-}Spc98}$ Pulldown His-Spc72$^{1\text{-}599}$ or His-Spc72$^{1\text{-}599,3R}$ quantification

IP_No.1

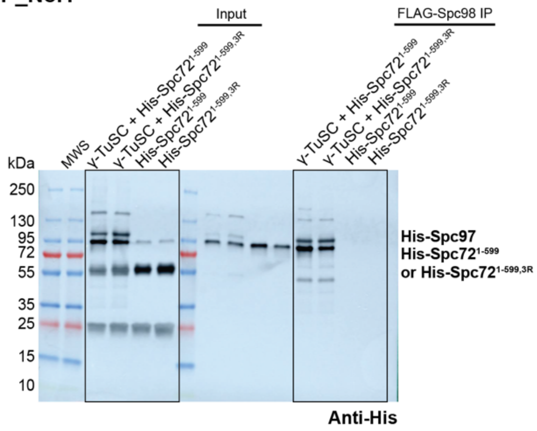

IP_No.2

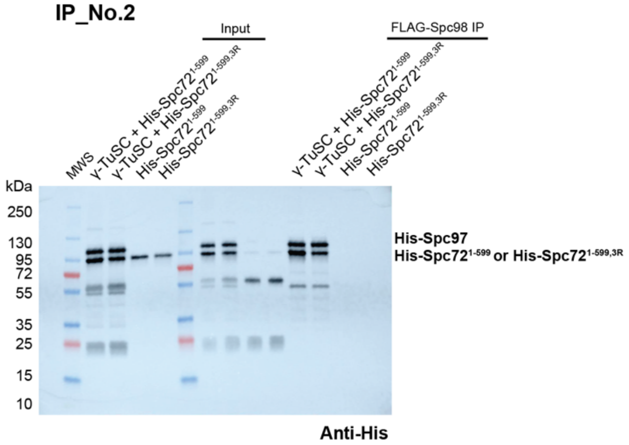

IP_No.3

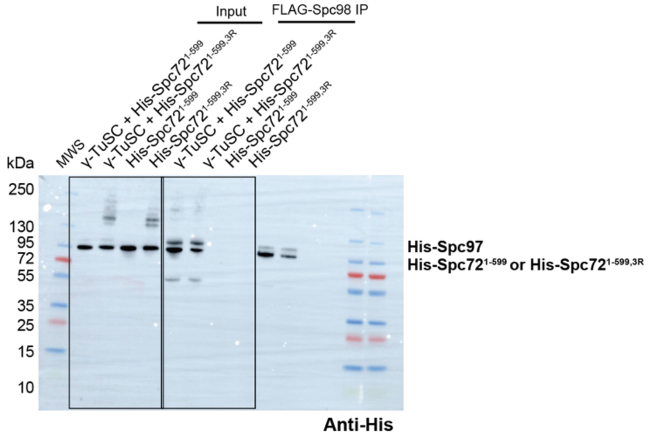

Source data, SDS-Page gels

Related to Fig.6e Stu2:Spc72$^{1\text{-}599}$ stoichiometry quantification from the SEC sample (fraction of 8.5ml)

Gel1, Supplementary Fig.1d

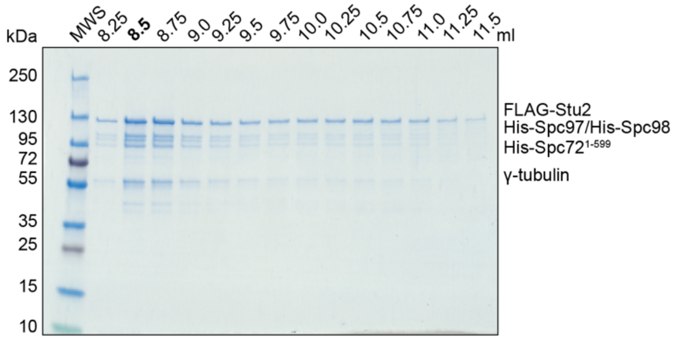

Gel2

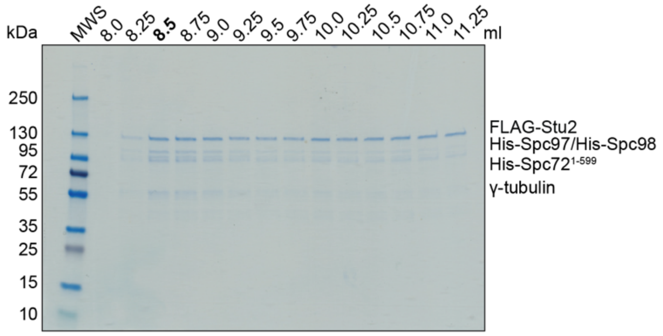

Gel3

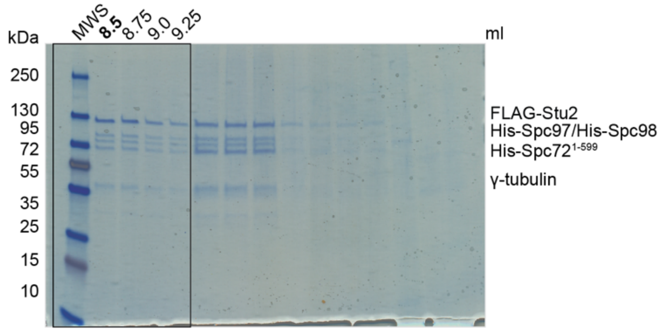

Supplement: Supplementary file 4 — Source data file [file 41467_2024_55778_MOESM4_ESM.zip › Uncropped gel and blot images.pdf]
